# The Spas of Germany

**Published:** 1842-10

**Authors:** 


### BRITISH AND FOREIGN MEDICAL REVIEW,

FOR OCTOBER, 1842.

### PART FIRST.

### Analytical and Critical Reviews.

### Art. I.

1. *The Spas of Germany*. By A. B. Granville, m.d. Second Edition. —*London*, 1838. 8vo.
2. *The Spas of England*. By A. B. Granville, m.d. Three Volumes. —*London*, 1841. 8vo.
3. *Pilgrimages to the Spas*. By James Johnson, m.d. — *London*, 1841. 8vo.
4. *The Mineral Springs of England*. By Edwin Lee, Esq., Surgeon. —*London*, 1841. 12mo.
5. *Memoranda on France, Italy, and Germany*. By E. Lee, Esq. Surgeon.—*London*, 1841. 8vo.
6. *A Practical Treatise on the Efficacy of Mineral Waters in the Cure of Chronic Diseases*. By Sir A. M. Downie, m.d.—*Frankfort*, 1841. 12mo.
7. *The Sanative Influence of Climate*. By Sir J. Clark, Bart. Third Edition.—*London*, 1841.
8. *Rapport sur l'Emploi des Eaux Minérales de Vichy dans le traitement de la Goutte, lu à l'Académie de Médecine*. Par M. Patissier.—*Paris*, 1840.
9. *Rapport fait à l'Académie Royale de Médecine sur les Eaux Minérales de France pendant les années* 1834-5-6. Par M. F. V. Mérat, rapporteur.—*Paris*.
10. *Theoretisch-praktisches Handbuch der Heilquellenlehre*. Von August Vetter. Two Vols.—*Berlin*, 1838.
11. *Marienbad, et ses différents moyens curatifs dans les Maladies Chroniques*. By C. J. Heidler, m.d. Second Edition.—*Prague*, 1841.
12. *Ueber den Gebrauch der Mineralquellen, im besondere derer zu Ems*. Von Dr. J. Vogler.—*Frankfort*, 1840.
13. *Kissingen ses Eaux Minérales et ses Bains*. Par F. A. Balling, m.d.—*Frankfort*, 1839.

14. *Ueber den Kurort Ischl.* Von Dr. E. KUNDT.—*Vienna*, 1841.

15. *The Baths of Creuznach.* By CHARLES ENGELMANN, M.D.—*Heidelburg*, 1841. 8vo.

16. *A Description of the Mineral Springs of Aix-la-Chapelle and Borcette.* By L. WETZLAR, M.D.—*London*, 1842. 8vo.

17. *The Spas of Homburg.* By Sir A. M. DOWNIE, M.D.—*London*, 1842. 12mo.

THE books whose titles we have transcribed, (and we could have added some dozens more,) and all of which relate to one subject—MINERAL WATERS AND BATHS—are of very various degrees of merit. Between the scientific treatise on the whole subject by the laborious German, who, with inexhaustible and untiring perseverance, seems to make himself master of all that every one has written on his favorite theme, for the purpose of laboriously framing it into a system ; and the thin pamphlet of the superficial spa-doctor, who extols with indiscriminating and excessive laudation, and with the clearest and most barefaced intentions, the virtues of the spring from which he derives his own means of subsistence, there are numerous intermediate books of very varied worth and purpose. Amongst them are not a few goodly-sized, handsome volumes, whose smooth, white paper, ample margin, and clear type, wood and stone and copper illustrations, and curiously-figured, many-coloured calico bindings, are one of the proofs that many opulent people in this country are bent on the endeavour to regain health, or to replace bodily uneasiness with more agreeable sensations, by change of climate, travelling, and mineral waters, instead of trusting exclusively to the ordinary medical treatment to which the majority are, with various success, most commonly submitted for the same purposes. As there is an increasing disposition, among those who are able to follow their own inclinations, to seek health by such remedies, these books are obviously well suited to the tastes and habits of society.

I. An author of the seventeenth century writes :

> "Oh England ! full of sin, but most of sloth,
> Spit out thy phlegm !"

Whatever our faults may now be, sloth is certainly not one : we have exchanged it at least for some others probably of the most opposite kind. Our natural characteristic is rather intense activity of the intellect, with corresponding energy in action, leading men to strive, at schools, for prizes ; at colleges, for exhibitions, fellowships, first classes ; in political life, for place and power ; in professional life, for distinguished station, founding families, great wealth ; in commerce and in trade, to outstrip rival nations and rival shopkeepers, and, if possible, to monopolize the commerce and manufactures of the whole town, county, province, or the world. The Americans include all this in the strong, unmistakable expression, "going ahead ;" and as a nation they illustrate much of the mingled good and evil belonging to this state of things : where life seems a race in which those who do not ever keep up with the rest, are run over by the hurrying, precipitating crowd, and are crushed. Our own profession strongly indicates this state.

Physiologically speaking, it is by the active intellect, by the mind—

or one part of it, rather—acting through the nervous system, that this general condition of things is worked out. The supremacy of the muscular system has long passed. "It is not (now) the thews and sinews of a man, but the spirit." In the patriarchal times, with the Greeks, the Romans, and with those they named barbarians, and amongst all whom we now call savages, with the barons of the middle ages, as well as their retainers, every man (except the inspired man—the poet and the priest) was, until the pen superseded the sword, greatly estimated according to his bodily strength, his mere animal man. But gunpowder, printing, and steam have strangely changed this. Muscular strength is alone estimated as so much mere machinery, and valuable only in the "hewers of wood and drawers of water," as worth so many pence a day. The men of strength are the men of intelligence, not the men of muscle. The acute, pallid, meager driver who directs the railroad engine whilst rushing on at its appalling speed, by a slight handle that can be moved by a child, is a type of this curious change. The pleasures of the day are similarly modified. Simple, unexciting enjoyments belong to a different state of society. "The child is father to the man," the early striving for rewards at schools is the first edition of the work of life—of the subsequent real struggle when the business of living begins: and this country is so thickly peopled, and sharpened, acute, practical heads are so numerous, that it is not to be wondered at, when the prizes are much greater and the combatants much more agile, that the contest should be severe. The man whose mind is always active in business requires stimulating pleasures. He can no longer be "pleased with a rattle, tickled with a straw;" but (like the Anglo-Indian who has been so stimulated by a tropical sun, that he cannot eat his rice and chicken without curry) his enjoyments must be highly seasoned, piquant, exciting. Not to speak of the more sensual animal excitements, such a man gets in his newspaper (full of those topics which are moving the whole world) a daily dose of the highest kind of stimulus; and in the succession of weekly, monthly, and quarterly journals, (which embody much of the literary ability of the day,) the more intellectual man finds the gin and beer of his jaded brain. To great and increasing numbers, politics are the condiments of their daily bread:—the news-room, the club, the liberal or the conservative association, holding their stated pothouse meetings, the inn-bar, a beershop bench, the town council, or the county townhall, are all arenas in which the affairs of the nation are discussed and settled with greater violence and much stronger personal feeling than by the chief actors themselves. The pleasures depending on the muscular system are comparatively few, and are still decreasing—practised almost exclusively by a few of the highest and, as extremes meet, of the lowest orders of society. By the former, hunting, shooting, and other field-sports are carried on with great perseverance: the same ardour which in the middle ages would have sent the same man to fight the Saracens in the Holy Land, to win the honours of ladies in tournaments or in perilous adventures abroad, to defend his castle against another chieftain, or to make vindictive or predatory excursions in the neighbouring domains of his foe, is now expended in carrying him over five-barred gates, through or over thick hedges, across forests, bogs, fields, on fleet horses, in pursuit of foxes or deer. Instead of books of chivalry, in which the adventures of armed knights in distant lands, and their combats, and various perils in pursuit of some imaginative idea are depicted, we have books of sports describing the leaps of the noble huntsman; the head of game slaughtered by the peer; the privations and hardships the patrician deer-stalker undergoes in wading streams, and creeping along the heather upon his belly, and watching for days his prey; or the arms and accoutrements, the boots and the coats with which some gallant colonel provides himself whilst waiting night after night in the depths of winter for the flocks of wild ducks which are to be the victims of his pleasure.

Amongst the poor there are a few muscular delights remaining: cricket, hockey, quoits, skittles, make up the poor catalogue of their out-of-door amusements. And by the multitudes whose bodies are fatigued with overwork, and whose intellects have had no opportunities for becoming expanded, is it surprising that the quiet luxuries of gin, beer, and tobacco should be so highly estimated? But the great bulk of the people—the middle classes, in its most extended sense, (including almost all who labour for their bread, except as servants, labourers, and mechanics,)—have no amusements depending on muscular exertion. In Scotland alone does it seem to be thought consistent with the gravity of years, with "respectability," with learning, for any one of this class to use their muscles for any other purpose than that of riding or walking. An Englishman sees with surprise on the ice of Duddington Loch, the principal of the university, the professor of moral philosophy, with perhaps a poet, an historian, a physician, a divine, silver or grizzle-haired men, employed with juvenile earnestness in rolling blocks of stone great distances, and with great art and strength ("curling," it is called:) an amusement which in his own division of Britain would appertain alone to boys or unoccupied young men. The Scotch, however, are in this respect much wiser, and, to a far greater extent than the English, combine active amusements with the strict performance of the more sedentary duties of life.

It cannot be denied that the chief pleasures of the day are of a similar character to its duties—mental, not corporeal. And as the brain and nervous system are the material organs through which all their objects are accomplished, it is not surprising that they suffer from over exhaustion, whether from over work or excessive pleasure. One of the most certain results of this activity being wealth, the luxury which is its attendant becomes in its turn a fruitful source of disease. The sensual pleasures which luxury supplies are often most keenly enjoyed by those whose nervous systems are stimulated by incessant exertion, and it is precisely to such persons that they are the most injurious. He who takes active exercise in the open air carries off many of the effects of a diet which under other circumstances would load his body with superfluous nourishment or stimulate him to excess; but the man who is engaged in a sedentary occupation, especially one requiring exercise of the active powers of the mind, eats and drinks too much with far less impunity: he is gradually and, to himself, imperceptibly sowing disease, and does not discover his mistake until his health is seriously impaired, and his habits become so fixed that often he cannot break through them. What has been said in a moral sense is equally applicable in a dietetic one:

> "At thirty man suspects himself a fool—
> Knows it at forty, and reforms his plan;
> At fifty chides his infamous delay,
> Resolves, and re-resolves, and dies the same."

The candle cannot be burned at both ends without double-quick consumption. The expenditure of nervous power required in active business—professional, political, commercial, or literary—renders the body less able to bear the labours of digestion, and to stand the depression which invariably follows the stimulus of too-exciting food and drinks. "Men dig their graves with their teeth" all the faster when, in addition to full diet, they neglect exercise by overtasking their brains. The consequence of such neglect of obvious rules is twofold. On the one hand, confirmed indigestion, and on the other, every form of nervous disease, both primarily arising from all those causes which exhaust directly nervous power, and secondarily from this exhausted nervous system sympathizing with an impaired digestion. The stomach, which even in the healthy state is the centre of sympathies, is now evidently more susceptible; so that whatever irritates it irritates the brain, and vice versâ. The stomach, which has been well but quaintly called "the conscience of a man's body," is now, like a conscience ill at ease, his torment. Our bodies being so framed that whatsoever they have been long accustomed to do or to bear, whatsoever has long acted upon them, either from without or from within, becomes almost necessary to them, it follows that our remedies in disease will be modified and changed in some degree according to the general habits and modes of life of the time. The epicure, the gourmand, the mere man of pleasure, the over-stimulated over-cultivated woman, the anxious care-worn professional man, the toiling, striving, struggling man of business, sooner or later feels that some "screw is loose," that the body has been too long trifled with, and that what is sown is about to be reaped. Whether it is called indigestion, liver-disease, hypochondriasis, abdominal congestion, exhausted nervous power, chronic rheumatism or gout, whatever local habitation the disease may have taken, or whatever theory may be adopted to explain it, the causes have been much the same—a neglect of organic laws of long continuance, daily irregularities in small omissions or commissions, bringing the body into such a condition that some more active, more apparent, and greater cause brings on disease. The patient himself is well aware that he has passed the Rubicon which separates him from health: he is conscious from his own inner sensations that he has an organized system, that he has a stomach, a head, or a heart, and that those vital functions which, like growth and life, had before been performed silently, unconsciously, and unerringly, go on so no longer. Perhaps the patient eats and drinks and looks well; it may be he is stout, portly, and fresh-coloured, and is often in fair spirits; and friends (healthy people, ignorant of what illness is) talk of imagination, fancy, nervousness, as if this theoretical explanation, this giving a thing a name, got over the difficulty. For what, after all, is more real than imagination, what more difficult to cure than its diseases, and what diseases (every madhouse testifies) more extremely obscure? In some cases the digestive organs are manifestly deranged; in others there are obscure symptoms of gout; in some deranged cerebral circulation; in some hysteria, convulsions, spasms, local pains, depressed spirits;—in all the patient is out of condition, not perhaps confined to his room, but uncomfortable in his feelings, and either quite unable to perform as he was wont the duties of life or only able to get through them with difficulty; or, if there are no urgent calls to exertion, is in a fretful, complaining condition.

To numbers who are in this state of health, victims, and, to certain extent, self-sacrificed victims of a highly-artificial and unnatural state of society, such means of cure as foreign and domestic SPAS, with the travelling and change they demand, offer a chance of relief which is eagerly sought after. Apart from the acknowledged virtue of mineral waters, and the advantages and pleasures which attend their use and increase their beneficial effects on the body, these spas are singularly well adapted to the wants, habits, and tastes of those who frequent them. For whilst the water acts medicinally on the body, producing healthiness and cleanliness of the skin, and giving moderate assistance to the secretions, and the change supplies the lungs with pure, fresher, and newer air, the watering-place at the same time affords EMPLOYMENT to the mind and body, and prevents or diminishes those sufferings from *ennui* which are almost certain to accompany the indispositions of those who have been used to an active life either of business or of pleasure. On this account also they are such agreeable remedies, so much sought after, for they supply a succession of stimuli of a healthy kind to those to whom from habit constant stimulus has become almost a necessity. In travelling, particularly in a foreign country, the mind is pleasantly excited to observation and reflection by a strange people, a new country, different styles of architecture, by works of art, or the beauties and sublimities of nature, by the novelties of manners, customs, costumes so widely varying from those to which it has been accustomed; the diet, the wines, the dishes, the hours are new, affording to the digestive organs a succession of new stimuli; and the air, instead of traversing the ocean, comes probably fresh from sweeping over vast continents, changing its character and its effects on the body exposed to its influence. . To the jaded man of pleasure, of business, or of intellect, a remedy which substitutes healthy excitements for the morbid ones to which he has been accustomed, must have great charms. At these mineral spas or watering-places constant employments are supplied him; his time is regularly divided, so that it slips away rapidly; and what with exercise to which he is compelled by the various daily water-drinkings and bathings at a distance from his hotel, his public meals, the interest in new faces and new manners, together with the various amusements which are at his disposal—all consistent with early and regular hours—he is, although an idle man (or woman), yet still a somewhat busy one. In such places in England, of course there is less stimulus to the mind; none of the pleasant varieties of foreign faces, costumes, cities; but still there is a similar effort to furnish employment and to turn remedies, as far as possible, into amusements. People are beguiled into exercise under the plea of promenading; they are compelled by fashion to rise early and to go early to bed; and in gay pump-rooms surrounded with all kinds of pretty decorations, do hosts of fashionable men and women daily for weeks with uncomplaining patience and unfailing assiduity, drink large tumblers of very nauseous water—doing in flocks what they would neglect to do alone, still chained by their habits, induced by fashion and companionship to take neutral salts, gregarious even in their diseases. Perhaps a fashionable physician is one of the attractions of the place; and it would probably be found that the assiduous way in which he employs his patients' time is an element in his success; mapping out for them their day's work, compelling them to live rationally and to take much exercise in the open air, and by water-drinking, walking, riding, driving, shower-bathing, and early rising, to fill up their time and drive away ennui. For the *dolce far niente* may be possible to an enervated Italian in his own luxurious climate, but its synonym is the Roman poet's "atra cura" to a people reared under colder and more changeable skies, to a life of active employments, or to one of as strenuous pleasures. No reader of Celsus but must be struck with the number of things which he gave his patients *to do*. It was no doubt for the wealthy and luxurious that he wrote, and the "gestatio in rheda," and the "clara lectio," and the hot and cold and vapour bathing, protracted and interchanged, must have taken up a good deal of time. So much bathing also with all its loiterings in the "sudarium" and other apartments of the bath, must have stood in the place, relatively to the skin, of a good deal of exercise, besides giving the invalid patrician occupation.

II. The medical evidence of the value of mineral waters in the cure of diseases is abundant even to profuseness in quantity, but its quality is indifferent. Indeed here, as in medical evidence generally, the quantity is in inverse ratio of the quality; for where little is known very much is usually said and written about it, and those diseases which are most obscure are most voluminously descanted upon. The books before us may be comprehended in three classes: the productions of casual medical visitors; the essays of the resident physicians; and learned and laborious systematic treatises.

1. The first class of writers are the offspring of the last few years, physicians who write for the general reader, and who combine with much popular medical matter more amusing details interesting generally. Such a writer in the few weeks in which he can escape from his private practice in London or elsewhere, instead of rusticating with his wife and family, as the more usual plan is, combines pleasure and business; with note-book and such writing gear crosses the channel and, by means of railroads, steam-boats, and the most rapid horse vehicles he can command, visits with British haste and activity every spa he can reach from Belgium to Bohemia, from the valleys of Nassau to the Swiss or the Noric Alps, and returns laden with his materials for a book. In this short time he has accomplished much: he has travelled rapidly many hundreds of miles (the exact number is usually told), he has studied all the natural scenery—rocks, woods, cataracts; "meadow, grove, and stream" have so fixed themselves in his memory as to enable him on his return to reproduce them with dioramic truth. He has examined with the eye of a connoisseur all works of art. Statues, pictures, cathedrals, monuments, ruined castles, battlemented towers, he has so justly valued as to decide on the exact place they are to hold in the estimation of mankind. As "the proper study of mankind is man," he has noted in his rapid course the physiognomies, manners, habits of the numerous countries he has passed through, and has been enabled to draw from his observations correct inferences as to the general character—moral as well as intellectual—of the various people he has seen, and to explain on the best principles of philosophy or political economy (as the case may be) the exact influence of laws, government, liberty or despotism, hot or cold weather, clear or foggy air, damp or dry seasons, in producing just the kind of people, and no other, as are found there. Or he may affect a somewhat lower range: he may examine the dress and manners of the "aristocratic," (a favorite term, although now so generally exploded, of one of these writers "qui stupet in titulis,") and decide on the exact scale of eminence which the spa holds by the visitors who frequent it, whose rank and dignity, disguised though it may be by green-sickness, gravel, gout, skin-disease, scrofula, or sciatica, is as clear as the day to his practised vision. Indeed, did he not appear so anxious to prove it, we should believe the travelling doctor was actually one of this favoured class, so entirely intimate does he seem with an "illustrious foreign prince," with "the Duke of This" and the "Marquis of That," with "amiable and most interesting noble ladies." At each medicinal bath and spring our informant, with a thirst for knowledge worthy of all praise, drinks the required quantity of fluid, whether impregnated with foul sulphuretted hydrogen, or holding in solution purgative salts, or fortified with iron; of all and of each—for the same watering-place may have waters of all kinds—he quaffs tumbler after tumbler, (with the quarter of an hour interval,) and then, with careful, discriminating self-examination, minutely registers in his diary the exhiliration and depression, the headach or intoxication, the purging, sweating, or diuretic flow which results from this self-sacrifice. He submits his body as well as his stomach for the good of science; and after soaking his fabric in every bath—hot, tepid, cold, in impure water, and in very filthy mud—he good humouredly and most enthusiastically dwells upon the delicious smoothness of his skin, its marble whiteness, the exquisite titillations of the small bubbles of carbonic acid gas which "just effleuraient the surface of the body," as they rise and burst by millions through "the lucid, genially warm, and gently murmuring" waters! The effects on his mind, the psychological as well as the physiological peculiarities are commented on with equal care, precision, and accuracy. One gentleman lying down in a tepid bath describes his feelings thus—"It is the human tempest lulled into all the delicious playings of the ocean's after-waves;" and we are told of water baths producing "the ecstatic state of a devotee blended with the repose of an opium eater;" passages of such poetic beauty and truthfulness as could have alone originated in some very peculiar source of inspiration, as if the "sacred brook of Helicon" were an aerated chalybeate water.

But besides this personal experience the observer introduces himself to the physician of the place, and often many pages are the result. Or else he masters the various pamphlets which their resident physicians have written and he prints the analysis and his own comments. The whole work is much enlivened by piquant anecdotes, personal remarks, quotations, classical allusions, French slang and phrases, and often the minute and highly-interesting details of the journey. Quarrels with rail-road clerks, ire at over-charged luggage, heartburn from over-eating at a cheap and well-furnished table d'hôte, invitations to evening parties, (reprinting the card,) give a life and reality to the journey, beguiling the reader to proceed, whilst also they afford an insight into the author's own mind, such as, were he not his own chronicler, we should have been ignorant and unsuspicious of. In one of the books of this class the author has been irritable and mightily irate with the editor and contributors to this Review; but as he, giving way to unreflecting impulse, expressed his momentary feelings of annoyance at the treatment he received at the Birmingham railway station, and in the next volume having grown cool, and seeing his error and his hasty conclusions, qualified his charges, so we will hope that in this instance time will also have made him wiser. At any rate, he cannot make us angry.

Among these popular works, the most remarkable is the small volume, by Sir Francis Head, which brought the spas of Germany into fashion. A man of genius is overworked, and sent by his physician to an insignificant spring in a small German principality; like any other traveller he wraps his cloak round him on the deck of a steam-boat, crosses the channel, ascends the Rhine, takes up his quarters at the Spa boarding-house, eats his meals at the tâble d'hote, bathes, and drinks the waters, talks with the bathman, and observes the visitors, wanders through the adjoining woods, climbs the hills, watches the swine, goes to church, and like the common herd of travellers he passes on and is unobserved; but not unobservant. Nothing has escaped his quiet eye: he has noted it all in his memory, and has found food for fresh and original thought in the commonest every-day occurrence. He returns, and writes a small volume, gives it a quaint title, ironically calling it a *Bubble*. Every one reads it; it is the theme of common talk, of universal praise. Every one has been in Nassau with the writer, has admired the fine English lads in the steam-boat, and has felt shame at the contrary impression the dandy gives of the national character. The baths, the snakes, the forked street of Swalbach, the swine, evening coming on, and the village suddenly lighted up, the solemn sermon and the serious hearers, are scenes fixed in all our minds by a power which by means of mere words, places before us pictures as vivid and real, and more exacting than a painter's. And a mere sportive *jeu d'esprit*, in which it has pleased the writer "ridentem dicere verum," sends hundreds, nay thousands, to an out-of-the-way village in Germany, to compare their own impressions with his descriptions, to see those things which without his aid they could have never seen, or like him to endeavour to renew their youth. And the dull and prosaic go there—and see nothing, and in vapid phrases accuse the "old man" of exaggerations and misstatements, priding themselves on their own foot-rule accuracy, ignorant perhaps that "the eye only sees what it brings with it, the faculty of seeing," forgetful how "different was the effect of the same objects on the retina of Newton, and Newton's dog Dash."

When such a vein has been opened, the workmen who press forward, endeavouring to quarry in the same direction are not few. Dr. Granville visited all the spas of Germany, and by a work written for the general reader, but containing much that interests the medical man, increased the common interest excited by the subject. Uniting the information of a book of travels, a "Hand-book of Germany," with much medical matter as to the chemical nature of the springs, and their curative action collected from books, conversation, and personal inspection, Dr. Granville's work has been serviceable in attracting the attention of the public to the valuable mineral waters to be found in that part of Europe.

"Pilgrimages to the Spas," by Dr. Johnson, followed Dr. Granville's two volumes. It is written with the same two-fold object, being both a book of travels and a book of science; the medical part, however, being much less adapted for the general reader than that of Dr. Granville's book, as it consists principally of brief and condensed analyses of the essays written on the springs that were visited, with critical comments. The work is characterized by that activity of mind, disposition to reflection, great industry, power of compressing the thoughts of others into a short compass, judicious practical knowledge of medicine, love of quotation of verses, and partiality to Apollo, Cupid, Minerva, Morpheus, Bacchus and other heathen divinities, which mark the productions of the same untiring, never-failing pen.

Mr. Lee, who resided some time at Wiesbaden, and has visited most of the German spas, has published a smaller work upon them, marked by good sense, reflection, and an acquaintance both with the action of these waters and what has been written upon them.

The favour with which the public received the "Spas of Germany" induced Dr. Granville to visit rapidly the spas of England, and to write three volumes about them. They are even more adapted to general readers than his former work; a table at the end of each volume, containing the analyses of the various springs, is the part which chiefly interests the medical reader.

2. The second class of books are the productions of the physicians resident at the springs. When Touchstone excused his love for the homely wench he had married, with "A poor thing, sir, but mine own," he uttered an aphorism embodying one of the causes which makes us doubt in admitting without hesitation the evidence of all very interested parties. It is the expression of that tendency of the human mind which leads the individual (for very wise purposes) to put an infinitely higher value on whatever belongs to himself, and that makes him attach so much more importance to the one subject on which his thoughts are occupied, than comparatively it deserves. Added to this actual bias the spa-physician has also his own temporal interests so closely bound up with the number of visitors who may seek benefit under his direction at these sources of health that his judgment may be somewhat influenced. Indeed, there seems to be something of that sort of anxiety in many of these gentlemen to include as many diseases as possible in their list of curable ones, as advertising quacks evince in newspapers and pamphlets to set forth the unity if not of disease, at least, the singleness of the remedy. On these accounts, books in general on mineral springs are among the most unsatisfactory, puzzling, and confusing of all medical or pseudo-medical productions. Infinitely tedious are the lists of diseases, the tape-worm prolixity with which they are discussed, the theoretical explanations of their action, and the discordant testimonies of each observer. But besides these empirical puffs, there are essays written by men of science and character, the books themselves testifying to the sense, judgment, and candour of the writer; and as these alone supply us with valuable practical matter, we shall chiefly use them.

Amongst these monographs, one of high reputation is the classic work of Marcard, on the waters of Pyrmont, written about 1780. He was a physician of Hanover, a severe sufferer himself from a nervous disease, which was cured at Pyrmont, but was again brought on by writing his book. The diligence with which of late years pathological anatomy has been studied by directing attention exclusively to changes in individual organs and structures, has led to the comparative neglect of those general conditions of the whole frame on which some of these local disorders depend, and on a knowledge of which is often founded a judicious plan of treatment. The study of the symptoms of general debility of the whole body; of weakness of the solids; of a depraved state of the fluids, showing itself constantly in eruptions, deposits in the urine, &c., of morbid irritability of the whole nervous system as testified by increased mobility, and sensibility, and mental sensitiveness; of accumulations of blood in the vessels of the abdomen, &c., are some of those circumstances which, although recognized by men who are successful practitioners, are insufficiently treated of in systematic and elementary books, but form the subject of many of the chapters of M. Marcard's volumes. In a main point these volumes differ from the whole class. The Pyrmont water is a strongly aerated chalybeate, and (as Marcard has written on the treatment of chronic diseases in general,) instead of showing how many may be beneficially subjected to this tonic and exciting treatment, he has shown, on the contrary, in how few cases tonics and strengthening remedies can be at first employed with safety, although there are many to be benefited by such means after having been subjected to a cooling, aperient, or alterative course. Marcard being himself an invalid, a practical physician, and one well acquainted with prevalent medical opinions, seems to have embodied the general sense of the ablest men of his day, confirmed by his own observation; and the same views have descended with some modifications, and are to be traced in the various volumes and pamphlets, and even in the showy semi-medical guide and tour books now so abundant. For those who are at all acquainted with the literary history of any subject, medical or general, well know that some single observer and thinker has furnished the staple matter that has been diluted, mangled, and deformed, or that has been illustrated and expanded by a host of succeeding scribes who have supplied books on the same subject. It was no small pleasure to discover among such as these a treatise of Dr. Heidler on the waters of Marienbad, a really practical book, full of judicious observations, and of the results of the careful study of the action of these waters on a large number of persons affected with different forms of disease.

Dr. Heidler is one of the best known resident physicians of any of the German spas, and is highly esteemed for his sound sense by all who are acquainted with him. His industry is untiring; he is frequently bringing out new works on this one subject, and his attention to it, his enthusiasm, and his common sense are conspicuous in all of them. That he may be somewhat partial to his own spa is an inevitable consequence in one who has resided at it a large portion of his life, and has watched its increase from an inconsiderable village to one of the most renowned watering places of Bohemia. With a due allowance for this bias we shall make much use of his work, as owing to the springs of Marienbad being of various kinds—aperients, alteratives, and chalybeates, Dr. Heidler's experience is very diversified; and as the two great varieties of waters, the saline and the iron, can be obtained at his spa, he is less exclusively wedded to either, and his results are applicable to waters of other spas which belong to the same classes.

The report on the waters of Vichy, made by the Royal Academy of Medicine of Paris, furnishes valuable information as to the action of hot alkaline waters on gout; and M. Bertrand's work on the waters of Mont d'Or in France affords materials for estimating the influence of the same remedy in chronic bronchitis.

Sir A. M. Downie, M.D. has written two little books. The first is to recommend Wiesbaden, and the second Homburg, a new spa. The plan of the first is good, and it is throughout sensible and judicious, but contains nothing very new. There is such a strange forgetfulness of opinions that our faith in the first was much shaken by reading the second. In the first, published in 1841, a description is given of the constitutional disturbance which is produced by the writers in much the same language as the native physicians employ; but instead of calling it a crisis, he prefers the term "point of saturation." The fact, however, is admitted that fever, discomfort, eruptions, and piles are not unfrequently produced by mineral waters after they have been taken for some little time, and that after these symptoms subside, "the full benefit of the course will be felt." In the second pamphlet, dated 1842, the same writer says, "during eight years that I have practised at the German spas, I have not seen one case of what is called crisis or 'bad sturm,' but admits he has seen symptoms of discomfort produced by imprudence in diet, exposure, and such causes during the course, or when the water has disagreed. Dr. James Johnson has the merit of converting our author. Between the publication of the two books, it seems that Dr. Downie had read Dr. Johnson's work, who theoretically imagines that a purge given before the course would obviate the disturbance which is thought to be "critical" and salutary.

The monograhs of Dr. Engelmann on the baths of Creuznach; of Dr. E. Kundt on Ischl, and of Dr. Wetzlar on the springs of Aix-la-Chapelle, have afforded us valuable information on these subjects. The latter book was needed; and will be found very useful to the visitors of the spa where the author so judiciously exercises his talents.

3. The third class are systematic works embracing the whole subject, in a chemical, theoretical, and practical point of view—compilations from the works of all other writers. Some of these are essential to those who thoroughly study the whole subject, as they are compiled with care, minuteness, and German diligence, by thoughtful, judicious men, who have themselves watched the effect of waters on disease.

Our object in this article is, to glean from these books the practical matter they contain, rather than to criticise very curiously their merits. The spas, particularly of Germany, are of so much interest at the present time, and the medical man is so frequently appealed to for information on their merits, and knows so little about them, that we offer no apology to those of our readers who may have more deeply studied their chemical history, for endeavouring, in a clear, simple, and comprehensive manner, to treat the whole subject so that the reader who has neither the time, inclination, nor books to enable him to investigate for himself, may have a general view of the various spas most frequented, the characteristic peculiarities of their waters, and the diseases in which they have been found most serviceable.

III. It cannot be denied that much scepticism exists among medical practitioners in this country, as to the practical efficacy of mineral waters in the cure of disease, and that patients themselves often take at least the initiative step in determining to try their effects. Such a scepticism is likely to be encouraged by the exaggerated accounts of the efficacy of these agents in so many different diseases, and by the difficulty of arriving at the exact truth from this host of partial and interested witnesses; and the medical man who is thus prejudiced, is the less likely to believe in the share which the mineral waters really may have in relieving his patients, when he thinks he can account for the improvement by the change of air and scene, freedom from cares of business or from the dissipations of pleasure, early hours, simple diet, and attention to the more obvious rules of health. But although these causes of improvement will explain much, and will sufficiently account for many cures, yet no one who will carefully read and reflect upon the chemical composition, and known action of those waters, (especially in Germany,) which are most active, but must admit that they are powerful agents capable of producing a decided impression on the secretions, excretions, vital and morbid actions of the body. The scepticism in this country must have been fostered by the fact that many of our waters whose virtues were most lauded and run after, differed very little from common water, and yet to their use was attributed most marvellous cures. Thus the hot wells of Clifton, the springs of Malvern, Matlock and Buxton, were found on chemical examination to be remarkably free from any solid matter whatever, and to be only very pure water rather warmer (except Malvern, which is cold,) than common water. And yet they were to cure consumption, scrofula, rheumatism and gout. A dislike, amounting to contempt of the marvellous, is a characteristic of the practical British mind; and in the case of these mineral waters the mere fact of the rich and the titled flocking to them, rather prejudiced the medical observer against them, as he knew it was that class who were the most easily seduced by anything new or wonderful. Their decline in public regard favoured the same unbelieving disposition. But on the other hand, Cheltenham, Leamington, Harrowgate in England, and more particularly many of the German waters, hold in solution such decided doses of medicines we are daily in the habit of prescribing, and their action upon the secretions is so palpable, that even the most sceptical must waver. Some contain active aperient neutral salts in such quantities as to act immediately on the bowels or kidneys; or alkalies which are sufficient to keep the fluids of the body in a constant state of alkalization: or such a combination of salts in small quantities, and gas, as to act immediately upon the kidneys or skin; or they are so highly impregnated with gases, such as carbonic acid, or sulphuretted hydrogen, whose action is felt by any one who will submit his body to the test; or they hold in solution the most satisfactory mineral tonic which we possess (iron), and in that condition, the state of carbonate, which all admit to be its most active form, and one which is retained in any medicinal preparation with extreme difficulty. Besides, many of the springs are hot, and the action of heat is no fanciful, hypothetical notion, but is one of the most obvious stimulants we possess. Its effects upon the cutaneous capillaries are marked; stimulating the minute vessels, and filling them with red blood, and thus tending to equalize the circulation, and to relieve internal conjestions, or irregular distributions of blood. The action of hot-water baths in producing perspiration need not be insisted on; those only who have seen the effects of the long-continued use of hot baths, even of the simplest volcanic waters, pursued *ad sudorem*, in reducing corpulency, are perhaps fully aware of the amount of visible power they possess. In addition to this effect, the body immersed in a hot bath at the time absorbs a considerable quantity of the fluid; thus a single bath of the hot waters of Vichy, which contain a considerable portion of carbonate of soda and carbonic acid, renders the fluids of the body alkaline; even of gouty persons whose fluids were previously acid. When therefore, in addition to the heat of these waters, we find they are impregnated with various active neutral salts, alkalies, iron, carbonic or sulphuretted hydrogen gases, &c., and they are applied daily and for many weeks to the whole surface of the body, which is capable of absorbing some of them, their therapeutic power must appear still more credible. It should also be recollected that these active medicinal agents are mixed in such proportion, that they act as aperients, diuretics, or tonics, (when they are suitable,) without any pain, uneasiness, or other discomfort, and that they are taken perseveringly, daily, for one or two months; the patient thus giving himself that chance of benefit from a remedy which he will rarely submit to when undergoing a course of medicine, for such a chronic complaint as does not entirely compel him to submission. How few patients, for instance, with any form of indigestion, or with chronic rheumatic pains, will second his medical adviser in the same way as he would do at a German spa? where he willingly assists the curative action of the medicinal water, by following the prescribed regimen of diet, habits, and hours, and giving up everything at the time for the sake of gaining health.

To doubt the power of mineral waters because they are not alone relied on, but diet, regular and early hours, exercise, &c., insisted upon in conjunction with them, is unwise, because in the cure of chronic diseases, (even by medicine,) the minute attention to these causes influencing the general health is essential. The conjunction of suitable medicines with hygiènic rules is the best treatment, but if either of them be adopted singly, medicine had better be altogether thrown aside, and care be alone paid to those agents, (air, food, clothing, habits, exercise, and cleanliness,) which are constantly acting upon the body. Such a doubt therefore can never be solved, is not indeed capable of solution, as both of these agents must act in conjunction in order that each may produce its due effect.

We are perhaps too apt to confound in our mind our own suppositions as to the action of the chemical ingredients which are found in mineral waters, and the actual and visible effects they produce on the body; and as we cannot explain an action, overlook the fact that it does really take place. But it is surely unjust to disbelieve evidence, that a water which contains little besides carbonic acid gas and a minute quantity of iron acts on the kidneys, because its chemical ingredients are not in our estimation diuretics. It is very fallacious to attempt to estimate the action of medicines on the body by reasoning prior to experience, however logical and clear the chain of reasoning may be. Occasionally analogy favours us, but the safest plan is to begin by experience, and then to reflect and reason on the facts which experience has furnished. Thus the virtue of some of our best remedies has been discovered either accidentally, or the medicine has been brought into use because the natives of the country (perhaps savages, villagers, unlettered and often unreasoning people,) have been long in the habit of employing them for the same complaints. Indeed, it is by far a safer way to seek information as to the virtue of any new remedy from the local tradition of the spot, than to apply it even according to analogy, which is the kind of reasoning that is most safe. By local tradition among the poorer class have the virtues of most springs been first discovered. It has been found that the people in the neighbourhood of springs have been well aware of the kind of diseases which were likely to be relieved. It is surely unreasonable to disbelieve this kind of experience, particularly when confirmed by the more educated and scientific who have subsequently confirmed it, merely because chemical analysis does not discover any active ingredient according to our notions to account for the effect satisfactorily; and particularly so when it is admitted that chemical analysis, though brought to such comparative perfection, is unable to detect the principles which produce contagious or epidemic diseases. If chemistry fail to detect the poison which in marshes produces ague, and in prisons typhus fever, in certain districts plague and yellow fever—poisons which we know to produce the same powerful effects in every human body exposed to them under certain circumstances, depressing the powers of life, setting up diseased actions, perhaps in a short time rendering diseased all our fluids, and often at once destroying life—we cannot wonder that it might also fail in discovering agents which in combination with water are capable of producing very salutary changes upon the body. We must go to experience first: we may then reason. When men who plume themselves on being practical men are prejudiced, they are the most confirmed theorists we know: they have been unused to reason, and when they attempt it they show themselves entirely incapable of estimating the weight of probable evidence—that evidence with which medicine has to do. The moment a mere practical man (so called) gets out of his narrow circle of individual observation he is lost; and such are those who are most disposed to doubt the efficacy of mineral waters: for here they leave experience, they have never had experience in their use; and they reason *à priori* from the effect of their own prescriptions; and as these waters do not contain the same doses, they doubt their power. The best chemists of France, celebrated as they are, have admitted that analysis has not enabled them to discover in many of the waters, whose effects on the body are well marked, the active principle; but instead of doubting the truth of these effects on the body, they have concluded that the failure only proved there were many bodies which escaped their means of examination. Chaptal said frequently and forcibly, that in the laboratory of the chemist, the dead body of the water was alone acted upon.* The

* Also Vauquelin, Annal. de Chimie et de Physique, xxviii., 105, and Robiquet, Journ. Pharm. 1835, p. 183.

commission appointed (under the direction of government) by the Académie Royale de Médecine, to report on the mineral waters of France, in 1836, declare their conviction that chemical analysis has not yet added in any considerable degree to that knowledge of the therapeutical effects of mineral waters which the experience of their effects had proved before chemistry was brought to their elucidation;* and they insist on the importance of taking into consideration the information which the inhabitants of the spot can furnish, "car il est remarquable combien les habitans sont instruits des véritables propriétés des sources qui les avoisinent, à tel point que parfois cette science populaire en dit plus que les livres à leur sujet."

We have said before that we must distinguish between our own notions as to the action on the body of the chemical ingredients, and the actual and visible effects which these waters possess. Now, although in many of these waters chemical analysis cannot detect the active principle, yet they do actually produce upon the body a visible effect: for instance, all the natural hot waters must produce perspiration; they must be absorbed; and they almost invariably act upon the kidneys: and therefore although their chemical composition may be simple, yet their action is marked, and in most cases sufficiently accounts for the relief. It is no objection against them to say that simple common warm baths would produce the same cures with the same attention to diet and regimen; they *possibly* would; but let the objector persuade his patients to make the same trial at home with his own baths, and furnish a series of cases to prove his point, and not be contented with a bare assertion, hitherto theoretical. As our object is to confine ourselves almost wholly to the action of these waters on the body in curing disease, we shall particularly insist upon their visible effects on the bowels, kidneys, skin, and on other secretions.

But in addition to the action of these waters as purgatives, diuretics, diaphoretics, and tonics, many physicians who have watched their actions are persuaded that they often act as alteratives, restoring healthy actions, and removing diseased ones, without any other effect very obvious to the senses; the improved health being the only visible change. But although many admit this alterative action yet they consider it is conjoined with some more visible one, and that usually during the course of treatment a crisis takes place; that there are symptoms of febrile irritation, uneasiness in the head, oppression in the breathing, strong and accelerated pulse, white tongue; and that these are the precursors of critical evacuations from the skin, bowels, &c. The knowledge of physicians of the action of alteratives is very limited: we recognize the effects only, but the means by which these effects are produced are unknown. In iritis, for instance, we see that lymph is gradually absorbed under the use of small doses of calomel and opium, which produce no sensible evacuation; bleeding and purgatives will produce the same effect very often, but at a greater expense to the constitution. Most of our alteratives, if given in larger doses, produce sensible excretions, (for instance, calomel, antimony, hydriodate of potash,) and it may happen that even in smaller doses they produce the same kind of action in a less

* Rapport fait à l'Académie Royale de Médecine sur les Eaux Minérales de France, 1834-5-6, p. 53.

evident manner, differing in degree only; both curing disease on the same principle,—aperients and other evacuants increasing the secretions more actively, and alteratives more gently. However this may be, we are certainly too ignorant of the *modus operandi* of some of the commonest medicines we daily prescribe, to have any good grounds for disbelieving, from analogical reasoning, the peculiar action of these waters. Analogy would lead us the other way: where the saline salts are in large quantities, they purge or act sensibly on the evacuations, and in smaller quantities when absorbed (as they are most readily into the system) there seems no grounds for disbelieving they can act as alteratives. Mineral waters may therefore be classed with those therapeutical agents, which act (1) as evacuants, producing secretions from the skin, kidneys, and intestines; (2) as alteratives; and (3) as tonics. The alterative action is, however, so generally conjoined with one of the others, that although we may recognize it, yet, for practical purposes, the twofold division, into evacuants and tonics is perhaps sufficient. Few practitioners of medicine but will acknowledge that they are most successful in curing chronic diseases, especially those in which the abdominal viscera are in any way involved, by producing visible secretions, and that tonics (notwithstanding their name) cannot be depended upon, and besides, are rarely prescribed, unless they have been preceded by aperients, or given in conjunction with them. In very many cases the judicious combination of aperients and tonics produce the most satisfactory results. It is in the same great class of diseases that mineral waters are useful, and in many nature has combined evacuants and tonics, so that whilst they produce secretions from the bowels there is no diminution of power.

IV. Regarded in a practical point of view, and according to their chief constituents, mineral waters may be divided into *Saline*, *Alkaline*, *Chalybeate*, and *Sulphurous*.

Saline Aperient Waters. The saline aperient waters contain many salts, which we are well aware exert a very marked and visible action upon the economy. Of these there are sulphate of soda (Glauber salt), sulphate of magnesia (Epsom salt), and muriate of soda (common salt); and the water which contains either one or more of these as its principal ingredient, when drunk freely, increases the secretions from the intestines, as well as their peristaltic actions, and acts as a diuretic. In some cases, the Germans say, no aperient effect is produced for some time; but after taking the waters for a fortnight, more or less, the body becomes saturated; general reaction, with feverish symptoms, comes on, which is relieved by some critical discharge. Waters containing one or more of these salts, and acting evidently on the bowels, are found at Carlsbad, Marienbad, Egra, Kissingen, Wiesbaden, and Baden-Baden, all in Germany; and at Cheltenham, Leamington, and Harrowgate in England. Of these the waters of Carlsbad, Marienbad,* Egra, and Cheltenham* owe their chief action to sulphate of soda, whilst the waters of Kissingen, Wiesbaden Baden-Baden, Harrowgate, and Leamington are strongly impregnated with muriate of soda. In torpid and phlegmatic habits of body, where such a course of aperients is necessary, the latter which contain the more stimulating muriates are preferable.

* The Kreuzbrunn of Marienbad, and the Old Well and Montpelier Spa of Cheltenham.

Sulphate of magnesia is the active ingredient in the water exported from Seidlitz and Seidschütz: and muriate of magnesia is in large quantity in the Püllna water.

ALKALINE WATERS. The salts of lime, in one or other of their combinations, are found in almost all mineral waters, though not in general in large quantities. In Germany a considerable portion of carbonate of lime exists in combination with the active aperients of Carlsbad, Marienbad, and Kissingen, as well as of Püllna and Seidschütz. Muriate of lime is in combination with muriate of soda, &c., in the Wiesbaden waters, but it is found much more frequently, and in greater quantity in the English springs, particularly at Harrowgate and Leamington. Sulphate of lime is a constituent of the waters of Bath and Scarborough. Bicarbonate of soda is the alkali most frequently met with. It is in considerable quantity, and is the principal ingredient in the waters of Ems, Töplitz, Mont-d'Or, and Vichy; it is also combined with saline aperients in the waters of Marienbad, Egra, and Carlsbad. In England it is found at Harrowgate, together with common salt, and also in small quantities in some of the less-known Yorkshire springs described by Dr. Granville. Carbonate of magnesia is found in the saline waters of Marienbad, and Kissingen, in the alkaline water of Ems, in the purgative waters of Püllna, Seidschütz, and Seidlitz, and in the chalybeate springs of Spa, Pyrmont, and Schwalbach,—in small quantities in all. It can hardly be said to exist in the English waters. There are traces of it only in some, and an appreciable quantity has been found in one spring at Cheltenham.

CHALYBEATE WATERS. Iron exists in mineral waters in the most convenient form for acting on the body, dissolved in an excess of carbonic acid gas. It is combined in small quantities with all the aperient saline waters of Germany, thus giving them a mild tonic action; but when existing in greater simplicity in waters saturated with carbonic acid gas it forms a most agreeable beverage, which women of the most delicate constitutions, labouring under real debility of the body, in which the stomach partakes, can drink in considerable quantities, not only without nausea, (as is the effect of our medicinal chalybeates) but with pleasure and gratification to their tastes; the carbonic acid giving the water a sparkling briskness, a piquant flavour, and an exhilarating effect on the whole system, somewhat of the nature of intoxication. It is not unlikely that the carbonic acid with which the mineral springs of Germany abound, may be one of the agents on which their effects considerably depend, as the effect of carbonic acid in the stomach, or even on the skin is very marked.

It is a powerful stimulant of the nervous system. When breathed we well know it is rapidly fatal by producing spasmodic constriction of the glottis; but when taken into the stomach or applied to the skin it is a powerful stimulant. The immediate effects of champagne on the system differs from that of other wine. The sudden exhilaration, the immediate increase of spirits (so often indicated by the general flow of lively talk), which even a small quantity produces, is not precisely similar to the intoxication from other wines, which is a slower process and attended with subsequent depression: and the intoxication is not at all in proportion to the quantity of alcohol; for champagne contains but a small proportion of alcohol in comparison to common dinner wines: whilst the ordinary French and German light wines, which are of the same character as champagne as far as regards alcohol, but which are not effervescent, do not produce the same instant exhilaration. The union of the carbonic acid with the spirit may be the cause of the difference of effect. Women with susceptible nervous systems and general delicacy of organization feel this sudden exhilarating intoxication at once, but by diluting the champagne with water it is much diminished, although the same quantity is drunk. A similar class of persons, who are unused to stimuli, feel an exhilaration and increase of spirits from soda-water; and at Pyrmont, in Westphalia, the chalybeate water which is highly charged with carbonic acid gas is drunk by the country people partly as a medicine, but partly on account of the kind of intoxication it produces. To the union of carbonic acid gas with so many of the German waters may perhaps be partly attributed the pleasure with which they are taken, the facility with which large quantities are drunk at a time, without any feeling of distension, the sensation as if the whole body was permeated and pleasantly acted on by the fluid, the increased appetite for breakfast, and the improved spirits. We shall not soon forget the pleasure of drinking at its source among the mountains, after fatiguing rambles, one of these highly aerated waters, which was otherwise so simple in its chemical composition that to its carbonic acid chiefly could be ascribed its effects. In pharmacy also the grateful stimulus of carbonic acid is constantly recognized, and saline aperients in an effervescing form are the most agreeable medicines that are prescribed. The effect of carbonic acid gas applied externally in paralysis is great; (we shall return to this subject when examining gas baths.)

Sulphureous, Ioduretted Waters, &c. Some other principles which we are in the habit of administering as active medicines are found in mineral waters, such as sulphur, iodine.

*Sulphur*, so much used for piles, cutaneous diseases, rheumatism, &c., is found in form of sulphuretted hydrogen in the cold waters of Harrowgate, and in the thermal springs of Aix-la-Chapelle, Barèges, and some of the other Pyrenean spas.

*Iodine* has been discovered by chemists in a few foreign springs, as well as English ones. At Woodhall, in the neighbourhood of Ashby-de-la-Zouche, are springs, the water of which contains more iodine than has yet been found in any British waters. In a gallon of the Woodhall water there is as much as half a grain of iodine, whereas no more than one tenth of a grain has been hitherto found in Britain in the same quantity. (Granville.) Local interest has been excited in these springs in scrofulous and rheumatic affections. Creuznach is the chief iodine spa of Germany.

V. We shall now proceed to give a general view of the diseases which have been benefited and cured by mineral waters, in order to convey clearer ideas of the whole subject, and to prevent repetitions when describing the virtues of the particular springs. Instead of going through the whole nosology and introducing every disease which by any possibility may improve whilst the patient is undergoing the strict diet and excellent regimen which are observed at these watering places, we shall confine our observations to those complaints which both theoretically and practically, both from what we should judge *a priori*, from our own experience in the treatment of diseases by the ordinary method, and what we gather from the testimonies of those physicians who have had ample opportunities of closely watching the effects of these waters upon the human body,—appear to be relieved in a marked and indisputable way by this peculiar class of therapeutic agents.

As a general rule,—and all such rules in medicine must be construed generally, and never be supposed to be without exceptions,—mineral waters are inapplicable in all acute inflammations, in all the more active stages of chronic inflammations marked by pain and febrile action, in diseases of the heart, and in aneurisms, in epilepsy, in tendencies to apoplexy or great congestion of blood towards the head, and in dropsies. They are applicable to chronic diseases, and to such stages as are unattended with febrile action. From some observations at Vichy it would, however, seem that acute attacks of gout in the joints do not contra-indicate their use.

It may be said generally that mineral waters are especially applicable to the diseases of those who by over indulgence and luxury, by a neglect (voluntary, or from circumstances,) of that temperance in all things, which is one of the secrets of well-being, or owing to these causes combined with a faulty organization entailed upon them by the sins of their ancestors, suffer from derangement and disorders of the general health, or from local maladies depending on the same general causes. Such are diseases of the digestive and abdominal organs with their numerous train of sympathetic disorders;—nervous complaints, particularly such as are symptomatic and dependent upon disordered digestion, or uterine irritation, as well as a rarer kind resulting from pure debility;—gout and rheumatism, generally connected with a faulty digestion; and gravel, stone, &c., the diseases of the same disordered condition; skin diseases and chronic diseases of mucous membranes attended with undue secretion, and often (together with enlarged glands) connected with a strumous habit of body.

This summary certainly embraces a large catalogue of diseases to be cured or relieved by the same remedy; but it must be remembered that mineral waters include saline aperients, diuretics, alkalies, and tonics, besides acting (as hot baths) very powerfully upon the skin, by stimulating it and promoting perspiration. Thus they comprehend such remedies as in practice we prescribe most commonly, and find most efficacious in the majority of chronic diseases which we have to treat.

Although not disposed to value a theory of disease as more than a generalization, not absolutely true, but as including and explaining the class of facts we are at present acquainted with, which a more exact knowledge will probably displace for a more comprehensive theory, yet we would not reject the attempt, for we are all (though we may be unconscious of it, as was M. Jourdain that he had been talking prose all his life,) inveterate theorists. Upwards, through the different degrees of practitioners of medicine, from the "old woman" to the man whose practice has been guided by the most reflective and steady reasoning, all try to explain, to give a reason for the belief that is in them; and as this is the tendency of the human mind, and a very useful one, we shall introduce here in as short and as comprehensive a form as we can, the general explanation of disease which many of the most thoughtful and practical Germans now adopt, and which is, we think, a successful one as far at least as it points to a correct plan of treatment. We shall take for our text the view which is given by Vetter of the opinions of Puchelt, Kreysig, Sachs, and other German physicians, as well as his own; but not entering into it with the same minuteness, nor touching on the more speculative parts. The words which, in this country, are alone used by metaphysicians of the transcendental schools, and mysterious speculations on that most mysterious of all subjects,—life,—are commonly met with in German books of medicine, but are especially unsuited to the peculiar state of medical intellect in this country.—But to pass on to the theory.

The body comes into a plethoric state, the consequence of which is an irregular distribution of blood to some particular organ: an undue quantity is sent to it, overfilling it. Diminishing the quantity of blood by venesection will relieve this, but only temporarily; the same condition will return, requiring bleeding at regular and stated intervals, and, although the character of the disease may be changed, health is not restored. We must, therefore, look for the cause somewhat further on, and see whether or not the state of the digestive organs is not such as to supply imperfect blood. On very careful examination, the error will be found here, and, if discovered early, a strictly temperate diet, water-drinking, and the other hygienic remedies, will restore the threatened health. Few, however, are aware of the necessity, or are willing to submit to be deprived of present gratification with the expectation of future and distant benefit; and nature has many processes of her own to restore the lost balance. The activity of the liver is increased, and the flow of bile relieves the disordered condition of the blood, or slow dilatations of the vessels take place, forming diverticula for the plethoric condition, as varicose veins, or places for actual discharges, as piles. Every secerning tissue is ready to separate from the blood products of abnormal nutrition, according to its own powers. The cellular tissue often separates it copiously in the form of fat: hence unhealthy fatness so commonly precedes inveterate abdominal diseases. The disease now takes one form or another, according to the organ or tissue involved; partly as their actions are morbidly increased or diminished, partly as their substance is changed, enlarged, thickened, hardened, or filled with morbid products. This is the explanation of those diseases in which the blood is overloaded with fibrin. There are other classes in which the nervous system refuses to be an agent in carrying on the morbid changes, in which there is no harmony between the morbid processes and the wants of the economy; —or, we may state it thus,—that the vis medicatrix naturæ is altogether unable to cope with the disease, its regulating power is lost, and anomalous, irregular, incurable diseases follow.

If this theory is correct, and the steps of disease are, disordered nutrition, producing a blood abnormal in quantity and quality, from whence arises irregular distributions of blood to particular organs, and in time organic changes in those organs, it follows practically that we must look to the digestive organs as the great means of cure, and our endeavours must be to produce a more wholesome blood. In applying these views to practice, we necessarily come to consider the disorders of digestion in the first place, and first of the earlier stages of these.

In early dyspepsia, the tongue may be clean, the countenance healthy, the body neither ill-nourished nor unhealthy in appearance, neither the pulse, respiration, temperature, nor evacuations perceptibly wrong, still there is more or less loss of appetite, disinclination to food, inclination to nausea; or, on the contrary, too great desire for food, quick and unusual return of the feeling of hunger, the sensation of satiety takes place too soon, or is too long delayed. In other cases, together with these, there are more marked local symptoms: sensations of heat, heartburn, fulness, oppression both of the head and stomach, nausea, changes of taste, of smell. These symptoms may depend on the diet, and cease when it is changed; or they may be owing to irritation or to debility, as in commencing old age, and in pregnancy, &c.

When a disordered digestion has continued some length of time, other symptoms ensue, such as chronic vomiting or diarrhœa, acid secretions, heartburn, or diseases of the abdominal viscera from irritation and congestion. Sometimes mucous, gastric, or bilious fevers are developed under the immediate influence of atmospheric causes acting upon this unhealthy state of body. Or the primary symptoms of indigestion may subside, and the disease may be transferred to the lymphatic vessels and glands, or to the veins. The diseases in which the excitability of the lymphatic system preponderates over all others are the various forms of scrofula; eventually the venous system gradually predominates over the irritated lymphatic system; the veins are considerably increased and distended, whilst the lymphatic vessels and glands decrease in their relative size and activity.

The second group of diseases are those in which irritation of the nutritive functions is followed by a plethoric state of the veins. This condition of the venous system, although not sufficiently recognized in the English schools, was not overlooked by the older writers, who admitted "plethora ad venas." The Germans regard it as a condition with which many forms of disease are connected, such as local congestions of various organs, scirrhus, fungus hæmatodes, many varieties of nervous diseases, and hypochondriasis and hysteria, which depend on organic causes. Abdominal plethora, depending on congestion of the veins of the portal system, is, it is thought, a very common complaint, and one in which mineral waters of the aperient class are eminently useful. Forms of gout, stone, and venous dropsy are supposed to be in connexion with this same condition of the veins, which are imagined not only to be passively over-filled and dilated, but to be in a state of increased activity and irritation. It is not surprising that, having these views, the Germans should regard piles as so important an indication of the internal state of the abdominal viscera, and as often an efficient safety-valve for their diseases.

With such pathological principles, says Vetter, which are embraced by those physicians who have an eye to see the unity of idea behind the vast variety of appearances, a more extensive view is taken of the nature of disease and of the action of remedies; and the observer regards whole lists of names of diseases as pathological metamorphoses arising from one and the same cause. He ceases to wonder that the same remedies cure so many apparently different complaints. In fact, this is a comprehensive view of the constitutional origin of local diseases—a truth which Abernethy saw so clearly, whilst he was candid enough to confess that he had not attended to medical cases with that degree of observation which would lead him to clearly develope the whole treatment. His one remedy—a mercurial and a black draught—was an unfortunate one; not in his hands, perhaps, but in those of others who signally abused it. Abernethy stated that he never had had experience in purging: his method was, to excite by means of medicine some copious and healthy secretions; but his scholars went (as people usually do) much beyond their master, and were inveterate prescribers of purgatives, introducing a practice which, indiscriminately adopted, has been attended with much mischief. The practical application of a theory which traces so many diseases to a faulty state of the digestive organs, and grounds the basis of their cure on improved nutrition, will be more obvious as we enter a little more into detail respecting the nature and character of these complaints. To this we next proceed.

A. *Diseases and disorders of the digestive organs.* We have alluded to the early symptoms of these diseases, but it is rather their consequences for which mineral waters are sought; and the class of waters which produce the most benefit are those which excite copious secretions from the mucous membrane of the intestinal canal; their efficacy chiefly depending on glauber, epsom, or common salt, in combination with carbonic acid, and small quantities of the salts of magnesia, and a little iron. The symptoms of chronic diseases of the abdominal organs or of their secretions are, according to Heidler, a yellow, pale, or cachectic complexion, want of appetite, bitter or pasty taste in the mouth, vomiting, oppression or cramp of the stomach, colic, constipation or diarrhœa, menstrual irregularities, sterility, leuchorrhœa, together with symptomatic disturbances of the nervous system, as hypochondriasis, hysteria, epilepsy, headaches, giddiness, dizziness, tinnitus, want of sleep, low spirits, palpitations, languid muscular power, cold hands and feet, small, feeble, hard, and sometimes intermittent pulse. Abdominal plethora, that is, obstructions or congestions of one or more of the abdominal organs, (running into organic changes of structure,) from a morbid excitement of the vital activity of the veins of the abdomen or of the whole body, (*venositas aucta, turgor venosus,*) is considered to be the very common cause of these symptoms. Stahl, Kaempf, Koch, Marcard, Kreysig, Hufeland, and other eminent physicians of Germany, who have recognized the frequency of this condition, have recommended aperients and deobstruents that do not weaken. This is the effect of the saline aperient mineral waters of Germany; such as those of Carlsbad, Kissingen, and Marienbad. In such cases, Dr. Heidler says, the Kreuzbrunn of Marienbad produces morbid evacuations of the bowels of every kind, green, black, gray, glassy, gelatinous, like pitch, or yelks of eggs, or ley, and coagulated blood; and this evacuation may be continued for weeks with a manifest improvement of strength, appetite, spirits, and a return to health. The brown and earthy tint of the complexions of many patients becomes clear, and their dispositions more gay and natural after having evacuated for many weeks or even months black excrements like pitch, by the use of these waters. Dr. Heidler adds, that the existence of this complexion should always fix the practitioner's attention in chronic disease; for even when it is the result of some preceding disease, it may, before its elimination, contribute to and aggravate the symptoms.

There can be little doubt that stools of this kind are rather excrementitious matters discharged from the system than mere accumulations in the intestines which are brought away—a fact recognized by the ancients who attributed many diseases to atra bilis, as well as by many moderns. Ramazzini considered that a general sallowness and darkness of skin, with a torpid condition of the skin, and faulty digestion, in workmen who led a sedentary life, was owing to the excrementitious parts of the fluids being imperfectly discharged. The most marked examples of abdominal plethora in patients at these spas are fat, abdominous, middle-aged Germans, who live sensual, sedentary lives, eating and drinking gluttonously and smoking incessantly; they resort annually to their spa, requiring without doubt to be cleared from that load of excrementitious matter which their habits have accumulated, and which Dr. Heidler so graphically describes as expelled from their bowels for months together.

But the same disease often exists in those whose external condition is very different, and who are suffering from various nervous symptoms which are often attributed to mere debility, and are treated accordingly. The sallow or pale colour of the face is not an invariable symptom. Our countrymen who are of fair complexion, who live on succulent meats, and are much exposed to the open air, often get into this plethoric condition and suffer severely both from the local symptoms of indigestion and hypochondriacal and other secondary nervous symptoms, although their fresh and high colour, their "fair round bellies," and their portly forms seem to indicate high health. It will be found, however, that gentle saline aperients will bring away from these, similar secretions.

Dr. Heidler's theory is, that the saline mineral waters which increase the secretions from the intestines differ in their action from purgatives, with which they are often confounded. Purgatives act by their irritating and often poisonous properties. Nature gets rid of them, as of any other morbid irritant, either by increased secretion or increased peristaltic action. But this water [he refers to the Kreuzbrunn of Marienbad, but his observations are applicable to all similar ones] is not an irritant. The rapidity with which it is digested, its rapid and complete absorption from the intestinal canal, the pleasure with which it is taken, the total absence of any disagreeable sensation, and the feeling of health which follows the evacuations, as well as the quality of the excrements, all prove this. They are critical evacuations, the effect of the increased healthy action of the organs from the roused vis medicatrix naturæ, and altogether different from the increased activity—evidently morbid—produced by purgatives. These are rather deobstruents (restoratives); and by confounding their action with purgatives, a fear is engendered lest they should weaken and injure the stomach. But how can a remedy which increases the appetite and digestion attack the stomach? how can a remedy diminish the vital powers which developes the impaired muscular power, renders the circulation more free, the pulse stronger, the mind more calm, the sleep more tranquil, the complexion clearer? The fatigue sometimes felt by those who drink this water is like the lassitude of the plethoric, or that arising from a large meal, or from a glass more wine than usual. It is particularly felt by the sanguine and irritable, and arises from the slightly exciting nature of the water, and often from the simultaneous use of hot baths, or from too much exercise, or it is the forerunner of a crisis. But whilst we accept with satisfaction Dr. Heidler's facts as to the pleasant and agreeable way in which these waters act upon the body, so different, indeed, to our pills and senna draughts, yet we do not think there are sufficient grounds for removing them from the great class of aperients. Owing to Abernethy and Hamilton, the action of aperients has been more closely studied in this country than on the continent; and we well know that, as a general rule, aperients relieve most efficaciously when so combined as to produce the least pain and disturbance. Hippocrates in one of his aphorisms asserts the same thing, laying it down as an axiom, that a purgative has been given with judgment when its immediate effect is relief instead of discomfort. It is needless to state in this country, that jalap, colocynth, scammony, calomel, &c., may be so combined as to act without any griping feelings of irritation, and that they often, when judiciously persevered in, bring away large quantities of foul, fetid, unnatural excretions, with a feeling of improved strength, and ease, and with relief to very anomalous nervous symptoms. In diseases of children this is particularly the case. In such cases we have, like others, been long in the habit of regarding the stools as the result of the excretion of excrementitious matter from the body, promoted by the purgative through the channels of the liver and bowels, on account of the quantity, the long continuance, and the varied appearance, increasing often in unhealthy aspect as the purgatives are persevered in. It seems as if it were the common result of this class of mineral waters in cases where the general health is much impaired from long-continued errors in diet, and other bad habits resulting from various degrees of luxury, to bring away foul excretions with relief. But in practice we know that the same effects follow very frequently a well-adjusted course of saline aperients which are so combined as neither to gripe nor to debilitate. They may probably act less surely, and be less pleasant remedies than nature mixes in her vast internal laboratory, but on this account we would not remove the latter into a distinct class, but rather believe that sulphate of soda or sulphate of magnesia, when in natural solution, act very much in the same way as the same salts when dissolved by ourselves from crystals, or even like the judiciously-prescribed aperients from the vegetable or mineral kingdoms; which, however, are more dangerous tools. In cases, then, of diseases of the digestive and other abdominal organs of long standing, brought on by habits of indulgence in the pleasures of the table, or in long-continued errors as to quantity; in all persons the whole of whose fluids are disordered from such causes, and whose general health is deranged, the saline aperient waters are likely to be of use. Those which are most in vogue in Germany are the waters of Carlsbad, Kissingen, and Marienbad.

B. *Gout and rheumatism.* The theory of gout which explains most satisfactorily the symptoms and the effect of remedies seems to be, that it consists of some morbid principle or principles in the fluids of the body, generated either by hereditary predisposition or by a long-continued indulgence in stimulating food and drink, and in luxurious habits, or most commonly by both combined; producing, whilst circulating through the system, various anomalous and distressing symptoms,—indigestion, flatulence, low spirits, various nervous pains, palpitations, inflammation, and depositions in the smaller joints—chiefly of the hands and feet, acid sweats, deposits in the urine, &c. The connexion between gout and stone and gravel is well known: the same causes often produce both—nutritive drinks, rich meats, and all highly-azotized food; and in both diseases there is a tendency to the deposition of lithic acid in the urine. Gouty concretions in the joints are composed of lithate of soda; the sweat of gouty persons is acid, and according to Chelius there is double the quantity of lithic acid in the urine of such persons. Colchicum, it has been chemically proved, increases the same principle in the urine;* and according to M. Donné, excess of animal food, and the abuse of tea and coffee, and even tobacco-smoking produces crystals of uric acid; hence some chemical physicians have been led to regard the morbid principle of gout as uric acid. We shall recur to this subject when referring to the effect of alkaline waters on gout.

The following observations of Dr. Heidler are in accordance with the experience of every physician:

"The cure of gout does not consist in curing the crisis, (the fit;) the operations of nature should be then only watched, so that they may be neither excessive nor impeded. The patient often imagines he loses his disease at the same time as the pain, and exposes himself again to the bad influences of diet and air as soon as he is temporarily relieved, forgetting that this is the time to effect the slow and the radical cure. But unless using moderate diet, and abstaining from irritants, spirits, and all nourishment too abundant or of difficult digestion, unless giving up all those habits which distinguish the mode of life of the rich inhabitant of a city from that of a healthy and laborious countryman, all attempts to cure rooted inveterate gout are useless. The basis of the cure is, strict attention to regimen and diet. The patients should never forget that they carry with them the latent germ of the disease which is developed from time to time, and shows itself by inflammation of some joint; and unless the functions of digestion, chylification, sanguification, and nutrition, and the fluids themselves are improved, medicine is useless."

Thermal waters in the form of baths are those most esteemed in the cure of gout. Some of them are impregnated with saline salts and alkalies, but all, from their heat and stimulating action on the skin, promote powerfully the perspiration, and are of advantage in proportion as the abdominal functions are regular, and when nature has chosen the skin, kidneys, or joints for the excretion of the gouty principles (Heidler). From the acid nature of the excretions of gout, alkalies have been proposed as a remedy. Common alkalies were found to be inconvenient, as they disordered the functions of the stomach, unless given in very small doses: this effect was, however, obviated by combining the alkali with an excess of carbonic acid, and the bicarbonate of soda and potash were preferred. There are several mineral waters which, besides being hot, hold in solution bicarbonate of soda, and also are charged with carbonic acid gas, so that they are adapted to neutralize this acid state of the fluids, and to agree with the stomach: such are the baths of Vichy, Mont-d'Or, Ems, and Töplitz. MM. D'Arcet, Chevalier, and Petit have shown, by many experiments, some of them on their own persons, the rapid effects of the Vichy waters in rendering the urine alkaline. Two

* Dict. Univ. de Mat. Med. Par Merat et de Lens. Vol. ii. p. 360.

glasses (containing about 46 grains of bicarbonate of soda) render the urine speedily alkaline, and the previous acidity does not return for eight or nine hours. A single bath has the same effect; and, finally, those who drink as many as five glasses of the water, and bathe daily, have their urine rendered alkaline during the whole time they submit to the treatment, which is from thirty to forty days. This alkalization is not confined to the urine, but extends to the cutaneous transpiration and all the other secretions. This effect in the fluids, produced by bicarbonate of soda, has before been described by Falconer and others, also by Ingen-Houz. (Rapport, &c., p. 23.) In addition, therefore, to their heat producing copious perspiration, these waters must combat one of the most marked symptoms in gout, the acidity of secretions; and when due attention is paid to regimen and diet it is not surprising that they effect many cures.

Gout, however, is often combined with an irritable and congested state of the mucous membrane of the stomach and bowels, and with abdominal or general plethora; and, in such cases, the use of those saline aperient waters which increase the secretions from the mucous membranes of the intestines and from the kidneys, either before the hot baths or simultaneously, is strongly indicated. At Marienbad, there are both kinds of remedies, aperient saline waters and hot baths; and as gouty patients resort there from all parts of Europe the effects of the combination of these means have been tried on a considerable scale. The saline aperient spring, called the Kreuzbrunn, is a water which manifestly increases the secretions from the intestines and kidneys, and it first came into vogue from its beneficial effects in arthritic diseases.

"If experience," says Dr. Heidler, "is consulted as to those remedies which have had the highest and longest reputation for the cure of gout, they will be found to be hot baths externally, and mineral waters internally. Their action is to increase the activity of the organs of secretion, of the skin, kidneys, and intestines. Next to these come guaiacum, sulphur, antimony and other medicines, to which are attributed anti-arthritic properties, but these also, in small doses, increase transpiration, and all the secretions from the external surfaces, and in larger doses augment the secretions from the intestines and other internal organs. Further, experience teaches us, that among mineral waters the preference is due to those which may be employed without inconvenience in the greatest quantity, and for the longest time; that is to say, those which are of the easiest digestion, and pass most easily, increasing the appetite, and all the secretions and excretions moderately, and without disagreeable sensations."

Hot baths are often indicated in the more irregular forms of gout, in which "the gouty principle" is not expelled readily by the usual channels. It is well known that very ambiguous symptoms, such as hypochondriasis, irregularity in the actions of the heart, anomalous nervous symptoms, and various forms of indigestion, occasionally precede, for months, the first appearance of a fit of gout, or affect those who have previously had attacks of gout. In such cases our older physicians were in the habit of sending their patients to Bath to bring on a fit of gout, and if the waters produced this effect, the general symptoms were relieved.

"The following symptoms," says M. Petit, of Vichy, "indicate visceral or internal gout. When patients consult me with internal complaints of some standing, such as gastralgias, certain hepatic affections, sometimes accompanied with piles; some symptoms of affections of the heart or lungs, and if these affections have often varied in intensity, changed their situation, or ceased entirely to return in the same place—if they have resisted various means—if, finally, they are anomalous, unusual, 'bizarre,' I always enquire if their parents or family are gouty, and if they have been subject to gout, and if the answer is in the affirmative, I consider the complaint may be of a gouty character, and I do not hesitate to try the hot alkaline baths and waters, (especially if these patients have ever had pains resembling gout.) In the majority of these cases much advantage has been derived."

In *chronic rheumatism*, natural hot waters used as baths have been always held in great estimation. In the report to the Academy of Medicine on the mineral waters of France, from 1834 to 1836, by M. Merat, it is estimated that 30,000 persons, affected with rheumatism, annually resort to the use of the mineral waters of France, and with very decided benefit. Rigidity of the joints, (not anchylosis,) not of very long standing, a frequent sequence of rheumatism, is much relieved. In the selection of waters the general principles which would guide us in gout would decide the choice. If there was manifest disorder of the digestive organs hot water alone should not be trusted in. Dr. Heidler finds that the number who are cured of rheumatic pains at Marienbad is very great. There hot baths are combined with the internal use of waters which increase the secretions from the bowels and kidneys.

The connexion of stone and gravel with the gouty diathesis is well known. Renal and visceral calculi often arise from the same causes, and often alternate with gout, and the calcareous concretions in the joints, lungs, heart, and other organs of gouty subjects, are of uric acid. In such cases the hot alkaline waters of Vichy, Ems, and Töplitz, as well as the saline aperient waters which contain carbonate of soda and magnesia in small quantities, in addition to neutral aperient salts, such as those of Marienbad, are indicated. In a recent number we noticed, at considerable length, the effects of the alkaline waters of Vichy in such cases. As these waters rapidly alkalize the fluids of the body, they may reasonably be expected to produce somewhat of the same effect on calculi within the body, and are therefore suitable remedies in these diseases. In all such cases, however, it need not be stated that due regard must be had to the state of the digestive organs, and to the diet and habits, as alkalies can only palliate an effect, not remove its cause.

The hot springs which are in the highest repute for the cure of gout and rheumatism, are the hot alkaline waters of Vichy, Ems, and Töplitz; the hot salt baths of Wiesbaden, and Baden-Baden; the aperient salines of Carlsbad and Marienbad; the hot baths of Bath; the warm baths of Schlangenbad, Wildbad, Pfeffers, Gastein, and the tepid waters of Buxton, and some similar ones in England.

c. *Nervous diseases.* Under this head we here include only such complaints as are independent of organic change; those, viz., which are the result of pure debility, of a weak watery state of the blood, or are symptomatic of disordered digestion, and of uterine irritation, &c.

The effect of mineral waters in many (so called) nervous diseases, throws some light on this class of complaints. For instance, patients whose disorders have been treated ineffectually on directly opposite plans: with stimulants and tonics, with quinine, wine, electricity, &c., on the theory that their so called nervous diseases arise from pure debility, subject themselves subsequently to a six weeks' course of gentle saline aperients, which produce two or three loose feculent stools daily, and employ at the same time a simple unstimulating nutritious diet, with fit exercise and early hours, and with relief to all their symptoms. Such results must surely render it more than probable that the complaints of these persons did not arise from pure debility, from real loss of power; but were symptomatic of oppressed power, probably from a congested state of the gastro-intestinal mucous membrane from indigestion, congested liver, abdominal plethora, or some other faulty condition of the abdominal viscera.

In the few observations we have to make on this class of diseases, we shall begin with nervous diseases which really seem to depend on true debility, and shall then pass on to those in which the debility is only apparent. In so doing, we shall make much use of the observations of Dr. Heidler, who has evidently watched carefully and meditated correctly the effects, both of chalybeates and saline aperients in this large class of complaints. Indeed, such observations and experiences are of much use in the treatment of diseases by our usual remedies: they supply indications which, in prescribing, we often endeavour to fulfil, and with much benefit. In this point of view, the effects of mineral waters in disease are of much more general interest to the medical practitioners than is usually imagined. Patients, whose time and circumstances will allow them to visit German spas, are comparatively few; but those who have similar complaints to those relieved by these waters, are extremely numerous; and we all know how very rebellious to common remedies such complaints are, and what a source of anxiety they prove to the medical attendant; so that any hints as to a more satisfactory treatment cannot fail to be valuable. (We quote Dr. Heidler:)

"True nervous debility, depending on real exhaustion of the vital powers, arises from numerous causes, such as sensual excesses, hemorrhages, or losses of other fluids, severe diseases, miscarriages, or laborious labours, grief, long-continued or excessive mental application, want of exercise and pure air, too prolonged suckling, loss of rest, insufficient food, severe mercurialization. Such causes produce weakness of the mind and body, trembling of the limbs, impaired vision, hearing, taste, and smell, &c. The following are the symptoms of true debility when of long standing and uncomplicated: complexion pale, more or less sordid, thin and pasty; nose and upper lip often a little swollen, œdema below the eyes, but no other symptom of dropsy or scrofula; muscles flabby and soft, appetite unequal, thirst in proportion to the appetite, breathing quickened, palpitations with or without flushings in the head or chest at the slightest emotion or exercise; languor and general depression, pulse small, soft, and generally slower than in health, but sometimes quicker; frequent chills, cold hands and feet, secretions of kidneys and bowels often regular, sometimes diarrhœa and constipation, skin too much disposed to sweat or too dry, often general uneasiness, aversion to all exercise, as well as to mental occupation, temper sombre, pusillanimous, irritable."

For this condition the chalybeate waters, especially those charged with carbonic acid gas, are beneficial; that is, if there is still power of reaction and no dropsy, no latent inflammation, no organic visceral disease, no accumulations in the bowels, or congestion of the abdominal viscera, and particularly when it is evident that the causes before mentioned have produced the symptoms. Such chalybeates are those of Spa, Swalbach, and the Carolinen-brunnen, and Ambrosius-brunnen of Marienbad. But it is the opinion of Dr. Heidler, after observing the different effects of chalybeates and saline aperients in nervous affections, that true debility is but rarely the cause of the symptoms, thus confirming the chief principles of treatment so clearly and judiciously laid down by Marcard in the last century.

"The feeling of lassitude and exhaustion, a slow and small pulse, cold extremities and cramps, are usually only symptoms of sluggish circulation, plethora of the portal veins, and morbid irritation of the nervous system of the abdomen. The effect of remedies in these cases, as well as the history of the disease proves this. The symptoms occur in those who eat succulent meats, drink beer and wine, sleep much, are idle bodily and mentally. Those who gain hunger, thirst, and sleep by the sweat of their brow, and live on bread, water, and a little soup, together with the meat from which it is made, are not so affected. False or apparent weakness is common to many patients suffering from disorder in the abdominal circulation; their contracted pulse seems feeble, but it is not conjoined with the other symptoms which characterize either excess or want of vital power. Such patients have these symptoms: sensation of lassitude, palpitations and spasms produced by the slightest exercise and emotion, sometimes even by remaining a short time in a hot room, by a cup of coffee, a glass of wine, or by hot soup; they have constantly cold extremities, shivering, difficult and slow digestion, and complexion earthy, pale or bilious; sometimes, however, good, and indeed too red. Such persons are particularly women suffering from piles, congestions, or a high degree of abdominal plethora. They are believed often to be without strength and feeble, and are treated most injudiciously with tonics and stimulants."

The use of the saline aperient waters in these cases is attended with the greatest advantage. It is often the best antispasmodic and calmative, removing the cause in the intestinal canal by increasing its secretions. Its efficacy in nervous diseases which, under the most varied forms of hypochondriasis, hysteria, debility of the nerves, weakness of the stomach and intestines, make up so large a number of those who resort to Marienbad, both illustrates their nature and the best treatment. In all such cases, Dr. Heidler never neglects the careful examination of the abdomen, even when the patient makes no complaint and the digestion is good, and her stools regular. A fixed pain, slight tenderness on making pressure over the liver, mesentery, ovaries, &c., often are the symptoms of chronic irritation of these parts, the hidden cause of anomalous nervous symptoms.

"Experience shows," continues Dr. Heidler, "that chalybeates, as well as all similar remedies, whose principal effect is exciting and tonic, are less beneficial in these diseases than those whose action is to increase the abdominal secretions. Prudent physicians did not lose sight of this, even at the time when the fashion was to give tone and strength, and it was commonly believed that nothing was more debilitating than a purge, or an emetic, and when those who attended as much to the fluids as to the solids were ridiculed. The passing theory of Brown and Roeschlaub banished the old reputation of Carlsbad and other similar waters. There were never a greater number of hypochondriacs, never more complaints of abdominal affections and weakness of the nerves than when wine, quinine, iron and opium were the universal remedies. Zimmerman, R. Whytt, Tissot and others dissipated some of these illusions, and showed how generally nervous affections were symptomatic rather than local diseases of the nerves."

Chalybeates, as is well known in medical practice, are of much more marked efficacy in the diseases of women than of men. We can rely on them with more certainty in such cases than perhaps on any other medicine. The same must hold good in chalybeate waters, and therefore in chlorosis, amenorrhea, and in various hysterical or other nervous symptoms, which depend on a torpid state of the uterine functions, they are applicable. For impotence and sterility they are much resorted to on the continent. It is necessary, however, to investigate these cases carefully, for the debility may be more apparent than real. In many cases it may be advisable to employ both remedies, commencing with saline aperients, and finishing with chalybeates. At Marienbad, besides these two kinds, there is a third spring, (the Ferdinand's-brunnen) which contains less sulphate of soda than the Kreuz-brunnen, and more iron. This is often found useful in mixed cases, such as when the patients are phlegmatic and torpid, and, whilst requiring aperients can bear stimulants; or for the very sensitive and nervous, who are generally pale, and, although quick and sudden in their actions, are without real power. Such patients may have local debility of the digestive organs, such as are called cases of weak digestion and torpid bowels, the local condition being often produced by indigestible food, abuse of spirits, purgatives, emetics, and narcotics. Such debility is found by Dr. Heidler to be relieved by this intermediate water, in which a mild aperient and a tonic are combined.

D. *Paralysis.* This is a disease for which thermal baths are much used. Their benefit must depend on the causes, which are various and obscure. When the attack of paralysis is sudden and complete, rendering it probable that it is owing to an apoplectic effusion in the brain, benefit cannot be expected. Nature, we know, gradually absorbs or accommodates herself to the presence of the coagulum, and if the part of the brain in which it was effused, is not extensively disorganized and impaired, the patient slowly recovers the use of his limbs, until the occurrence perhaps of another effusion. In recent cases, the stimulus of a hot bath, by increasing the cerebral circulation, might be of great injury: when the disease is chronic, baths cautiously tried may perhaps be of some slight service in amusing the patient's mind, in affording him change, in supplying him with the most pleasant of all gentle stimuli, that of hope, while we subject him to a careful diet, and to a well-regulated hygienic plan; for in no other way, except in improving the general health, can we suppose benefit to arise. Dr. Heidler's experience of the effects of warm bathing in these cases is as follows:

"Paralyses, from apoplexy of many years' standing, which have not gradually improved, are incurable. The hope of cure is in proportion to the time and number of the relapses, so much the less as the intellect and muscles of the head are the more affected, as the abdominal functions are the more regular, and out of proportion with the paralysis, as the patient has been less bled at the commencement, and as he is more advanced in age."

Dr. Heidler has seen warm baths used in cases of from six months' standing to eight or ten years. Very rarely has he seen perfect restoration, and those who have not improved, have sought health in vain at other baths. But there are many cases of paralysis, even from apoplexy combined with congestions of the abdominal viscera, in which a course of saline aperient waters, combined with warm bathing, may be beneficial.

It should, however, not be forgotten that all mineral waters, when taken internally in any quantity, and also hot baths, have a tendency to produce congestion of the head, and therefore that where there is a tendency to apoplexy their use may be attended with danger and even death. Sudden deaths from apoplexy at the German's spas are not uncommon.

Paralysis coming on gradually is the most obscure, both in its nature and treatment. There may be no pain, merely gradual loss of power or sensation, slight loss of strength and diminution of the fineness of touch, which is attributed to weakness, and yet some organic changes may produce it.

"It is easier," says Dr. Heidler, "to attribute paralysis to weakness than to examine diligently whether the functions of the nerves are embarrassed by congestions of blood, varicose dilatations, extravasations, tumefactions, &c. This supposed weakness is especially dangerous when a plethoric and robust patient, with strong or suppressed pulse, complains of the first paralytic symptoms. The pulse in the diagnosis of paralysis is often of the highest importance. Whenever it is quick and hard stimulants are wrong even at an advanced period. Notwithstanding the appearance of debility, we should carefully avoid seeking the cause of an incipient local paralysis in the part itself, and using exclusively local treatment, even when at first the functions of the brain appear to be regular, and when there is neither pain nor swelling in the vertebral column."

Dr. Heidler suspects that many cases of apoplexy, called nervous or serous, may be wholly symptomatic of abdominal diseases; for in these latter affections there are many symptoms of depressed nervous energy, such as languor and muscular weakness. Might not, he asks, this condition be pushed further with complete loss of power?

Paralysis which, in Germany, is thought to arise from metastasis, and which, in this country, is overlooked, or disbelieved in (from our contempt of the opinions of our medical ancestors), is considered the form of the disease which is most likely to be benefited by mineral waters. The chief causes are supposed to be the sudden suppression of a natural secretion, as perspiration or menstruation, or of a morbid one of long duration, as certain skin diseases, leucorrhea, piles, &c.; and cases of paralysis of an arthritic or rheumatic origin are believed to come under the same class. In such cases of paralysis (says Dr. Heidler,) Marienbad has acquired its reputation; but when the disease is of many years' standing, and has resulted in idiopathic diseases of the brain and spinal marrow, or of the nerves, or with hectic fever, or great exhaustion of the vital power, a cure cannot be expected.

Paralytics rosort to all the natural hot springs, from the sulphurous waters of Barèges and Aix-la-Chapelle, and the strong hot salt springs of Wiesbaden, to the pure thermal springs of Pfeffers and Gastein.

E. *Chronic discharges from mucous membranes.* When leucorrhea, gleets, and mucous secretions from the rectum are complicated or dependent upon a congested or irritable state of the digestive organs, or a symptom of a bad, flabby, mucous habit of body, (cachexia pituitosa, or a strumous condition of body, in which there is a great tendency to mucous discharges,) it has been found that those waters which sensibly increase the secretions from the intestinal canal are very beneficial.

*Chronic bronchial discharges* are sometimes benefited by hot baths, used ad sudorem, producing a strong determination of blood to the skin. The hot alkaline baths of Mont-d'Or in France, under the judicious directions of M. Bertrand, the resident physician, are employed with much success in chronic pulmonary catarrh, and in chronic pneumonia, and in even hemoptysis in patients who are neither febrile, nervous, nor irritable, but whose habit is rather torpid.

Sir James Clark considers that a residence of one or two winters in Italy, and a course of mineral waters suited to the case during the summer, "afford the most effectual means we possess in the more obstinate and deeply-rooted cases of bronchial disease." The selection would depend upon the nature of the case, care being taken that the situation of the spa be suitable as well as the water, as it would not do to send a patient who could not bear a rarefied air to a spring in the Pyrenees, or one who was injuriously affected with damp to a warm close valley in Germany, although the waters might be peculiarly suitable to his case. When the patient's constitution is very delicate, and there are symptoms of abdominal congestion complicating the bronchial disease, Sir James Clark prefers the warm alkaline and slightly aperient baths of Ems.

"In cases of less delicacy, and those especially in which a mountain air promises benefit, or where the bronchial disease is complicated with chronic cutaneous eruptions, Bonnes or Cauterets (in the Pyrenees) will be more effectual. In cases where there exists a very torpid state of the system, and especially a languid or defective action of the skin, or where the occurrence of the bronchial disease has coincided with the disappearance of any cutaneous eruption, the system of bathing adopted at Mont-d'Or will, I believe, effect cures where other waters fail." (Clark, p. 349.)

F. *Chronic skin-diseases.* The obstinacy of this class of diseases to remedies, their liability to return, and to be aggravated by slight causes, need not be insisted upon; and, when they are of long continuance, the system seems to become so habituated to them that it is often a question whether it is not preferable to allow them to remain, than to run the risk that may be incurred by getting rid of them by active or powerful remedies. In medical practice a course of mild saline aperients, together with warm or tepid baths, and careful diet, are as successful in long-continued cases as any remedies we possess, and unlike arsenic, mercury, cantharides, and other powerful agents, can do no harm. We cannot doubt, therefore, that a course of one of these saline waters with warm bathing, change of air, and a good regimen is often serviceable. Dr. Heidler candidly says that his own views on these diseases are not clear. Many patients have been cured by a few baths; others have not been relieved by a large number. His experience makes him believe that where patients have chronic skin diseases of long standing, and are in good health, they gain no good, but when the affection is consequent on indigestion, scrofula, gout, &c., as is often the case, they are relieved by the waters which benefit these diseases.

Sulphurous waters have been especially esteemed in this class of complaints. The waters of Harrowgate owed their celebrity to their supposed efficacy, and they contain sulphur in union with neutral salts, in such a proportion as to act upon the bowels. Many of the patients who resort there have that red, pimply state of the face which is called "scorbutic," and which is often brought on by the sudden application of cold, or appears periodically, owing to a peculiar habit of body—but the waters also cure more obstinate and powerful cutaneous diseases, particularly when their internal use is conjoined with their external employment, as hot baths. The natural hot sulphurous waters of Aix-la-Chapelle and Barèges are, however, more efficacious than the cold sulphurous waters of our own country.

G. *Scrofula.* Regarding scrofula as a peculiar morbid condition of the whole system, often an hereditary disease, but constantly connected with, or brought into activity by a faulty condition of the nutritive functions, and susceptible of relief chiefly by a scrupulous and habitual attention to the diet, exercise, climate, and other hygienic remedies, together with a medical treatment of the particular local symptoms of the case, we can understand that warm bathing, and the internal use of waters containing such remedies as muriate of soda and lime, iron and iodine, together with others which increase and rectify the secretions from the intestinal canal, with change of air or climate, and a careful diet may often be beneficial. This class of remedies are not specifics against scrofula, and when it is deeply rooted and hereditary they are useless: they must be selected according to the symptoms of the individual cases. On the continent mud baths, and poultices of mud to the parts locally affected, are considerably esteemed, as well as warm bathing generally. "When the patient looks cachectic, and his face is swollen, his belly enlarged and pasty, or he has worms, mucous stools, dyspepsia, or constipation, or any other chronic affection of the abdomen, the use of such waters as the Kreuzbrunn of Marienbad, brings away large mucous stools, sometimes for months together, without weakening the patient, but on the contrary with an improvement in the appearance, strength, and health." (Heidler.) Chalybeates may be taken subsequently.

Such then are the groups of diseases for which mineral waters are employed, very various indeed in symptoms, and in name, but all more or less dependent upon or connected with a faulty condition of the nutritive functions of the body, and thus having a common point of union and of resemblance.

VI. We now pass to the consideration of the particular mineral springs; and as they are exceedingly numerous we shall notice those only which are most frequented, and particularly those of Germany. Our object will be, as far as we can, to give such a sketch of each as will enable the reader to gain a general idea of the nature of the water, and the diseases for which it is suited; our space forbids our entering into minute details, either of chemical analysis or of description.

A. *Saline Aperient Waters.*—Carlsbad. This celebrated spring lies in Bohemia, about 230 miles from Frankfort, and is, consequently, at the usual rate of travelling at five miles an hour, attainable only after a long and somewhat difficult journey. It is the most famous spa in Germany (the "king of the spas,") and is much resorted to by invalids from all parts of Europe. The situation of Carlsbad is very picturesque. There are several springs: the chief is the Sprudel. It is a hot saline water, a boiling spring (of the temperature of 167°), its chief active ingredient being sulphate of soda. It also contains common salt, a little carbonate of soda, and some iron, besides carbonate of lime; and it is charged with carbonic acid gas. It is employed almost wholly as a drink. Its action on the body is well marked, increasing the secretions from the mucous surfaces. From its heat, carbonic acid, and iron, it is stimulating, as well as aperient; hence it is efficacious in chronic stomach, and other complaints of the digestive organs in persons of a lymphatic and torpid, or of a bilious and spare temperament, who are not easily excited; also in those of a like temperament who have led indolent luxurious lives, and by eating and drinking too much have laid the foundation of deep-rooted affections of the abdominal viscera. It is, of course, contra-indicated in the highly plethoric and sanguineous, as well as in the irritable and sensitive nervous temperament. The crisis, or "bath storm," is here very marked.

The opinions of the Germans as to the efficacy of the Carlsbad water is very high. Hufeland, when asked by Dr. Granville the reason of the undiminished celebrity of Carlsbad, said: "C'est qu'il guérit des maux rebelles à tout autre moyen curatif." There is not a single medical man of eminence in Germany, adds Dr. Granville, who does not entertain the like opinion. The consequence seems to be that diseases of all kinds are sent there, so that a medical eye cannot detect any prevailing class of complaints by the appearance of the water-drinkers.

Kissingen. This spring lies in Bavaria, two or three days' journey from Frankfort. It is much resorted to by the Germans, and is also attracting the notice of English invalids. There are several springs containing the same ingredients, but in different quantities. The Ragozi spring is the most efficacious, and the one which patients visit the Spa chiefly to drink. Kissingen is a cold water, the principal ingredient being common salt (of which there are 62 grains to a pint); it is highly impregnated with carbonic acid gas and contains some iron. It is thus gently purgative, stimulating, and tonic. It increases the secretions from the kidneys and intestines. It was first brought into repute for its alleged virtue of curing sterility, for which it is still celebrated in Germany. In chronic disorders of the digestive organs, in the torpid and phlegmatic, in those who have exhausted their digestive powers by hard living, in chronic enlargements of the liver from similar causes, in sterility and other uterine affections, where the general symptoms seem to require iron in combination with a stimulating aperient, these waters are indicated. In the plethoric and those disposed to inflammation, as well as in those of sensitive nervous organizations, it is contra-indicated. The water of the "Pandur," another spring, is heated and used as baths. From its being highly charged with carbonic acid gas, it stimulates the vessels and nerves of the skin, and is said to be efficacious in some cases of paralysis, but contra-indicated in the plethoric or in those disposed to determinations of blood to the head. These baths must be a useful adjuvant to the waters taken internally, particularly when the system is torpid. Living is excessively cheap at Kissingen: a dinner for two persons, consisting of several dishes and some excellent Bavarian beer, costs one shilling and eleven pence. Every other repast is proportionably cheap. (Granville.)

Many of the other spas are resorted to as places of amusement as well as of health; this at present is devoted to its right object, and so strictly that "the tables d'hôte are under the strict surveillance of the authorities, and nothing is allowed to be served up that is likely to disagree or to interfere with the beneficial action of the waters." (Lee.)

Could some such wholesome despotism be exerted by the medical attendant over the table of his patients, he would not, in a vast number of cases, require to recommend a German spa; but men are now somewhat like what they were in the days of Naaman, the Syrian,—disdaining and getting wroth at the suggestion of a simple remedy near at hand.

MARIENBAD. This spring is also situated in Bohemia, about a five hours' drive from Carlsbad; it is comparatively a new spa, but much celebrated in Germany. Its situation is beautiful. It has several springs, both saline and chalybeate. The principal saline spring is the *Kreuzbrunn*, which is cold, containing a large proportion of sulphate of soda; it holds also in solution some common salt, carbonate of soda and magnesia, and iron, and is charged with carbonic acid gas. Hufeland regarded it as the Carlsbad water cooled, and on this account less exciting. It seems to be a water perhaps more generally applicable than any other. It increases the secretions from the intestines, kidneys, and skin; is easily digestible, and acts on the bowels mildly and without uneasiness, often bringing away for weeks together foul secretions with great relief. It is a combination of a gentle aperient, stimulant, and tonic. It is less purgative and exciting than Carlsbad, and less stimulating than the cold salt springs of Kissingen. It is, therefore, not contra-indicated like these in the sanguineous and plethoric who are suffering from disorders of the digestive and other abdominal organs; whilst it is often of great service to those who are apparently suffering from nervous debility, but in reality from a state of irritation of the gastro-intestinal mucous membrane, of which various nervous symptoms, as hypochondriasis, muscular debility, &c., are merely symptomatic. For the torpid and phlegmatic, Carlsbad and Kissingen are preferable; but in the sanguineous and the nervous, the Kreuzbrunn of Marienbad is the fit water. These three springs seem applicable to the same kind of diseases, but the selection should depend on the temperament of the patient, Carlsbad being the most stimulating, Kissingen next, and Marienbad the least. There are three other springs at Marienbad; two of these (the *Carolinenbrunnen* and the *Ambrosiusbrunnen*) are light chalybeates, highly charged with carbonic acid gas, like Swalbach, and often very useful after a course of the saline; or in cases of true debility. The third spring (*Ferdinandsbrunnen*) is intermediate, less aperient, and more tonic than the Kreuzbrunn, and not purely chalybeate like the two others. It is useful in mixed and doubtful cases.

FRANZENSBAD. This is the spa of Egra or Eger in Bohemia, and is a morning's drive from Marienbad. Its situation is dreary and it is going out of fashion, being superseded by the latter spa. It is a cold, highly-carbonated saline aperient; its chief salt is Glauber-salt, with some muriate and carbonate of soda and iron, and it is highly charged with carbonic acid. Franzensbad thus contains the same chemical ingredients as the Kreuzbrunn of Marienbad, but in less quantity; so that it seems to hold a middle place between the Kreuzbrunn, and the direct chalybeates, and may be more suitable to those of a delicate constitution, for whom more active waters may be injurious. The *Saltzequelle*, at the same place, is even less stimulating.

There are three aperient waters exported from Germany, and well imitated by Struve at Brighton. These are Püllna, Seidschütz, and

Seidlitz waters, in the neighbourhood of Carlsbad. *Püllna* water is a speedy and direct purgative, owing to its containing a large quantity of Glauber and Epsom salts. It also holds in solution muriate of magnesia. Wells are dug in the volcanic soil, and the water which soaks into them is bottled for exportation. Dr. Granville praises it highly; he had himself, like an Englishman, been addicted to the use of pills, but now, he says, it is only necessary to drink about two ounces of Püllna waters "to keep all straight."—*Seidschütz* water is of a similar kind, but, besides Glauber and Epsom salts, it contains nitrate of magnesia, which renders it even more bitter. It is an effectual purgative.—*Seidlitz*, the name of which is so well-known, is in the same district. The water owes its efficacy to Epsom salts, containing one drachm and two scruples in a pint. The Seidlitz powders have therefore nothing in common with real Seidlitz waters but the name and the effect.

In England the saline aperient waters are at Cheltenham, Leamington, Harrogate and Scarborough.

Cheltenham. The most active ingredients in the Cheltenham waters are Glauber and common salts; they also contain muriate of lime and magnesia, and a minute quantity of iron. The springs are numerous, and vary much in strength and in the relative proportion of the above-named ingredients. The common opinion is that the visitors are too numerous to be supplied with the real water, and that after a certain hour of the morning, an artificial water of the same character is substituted. The careful way in which the springs themselves are concealed does not tend to dissipate this common delusion, if it be one. The waters act upon the bowels considerably and regularly, without uneasiness or feeling of debility, and are thus of advantage in indigestion caused by full living, as well as in many complaints of the biliary system, brought on by the climate of India, a stimulating diet, and mercury. "The East Indian visits these springs, as a matter of course, upon his arrival in this country." (Scudamore.) "The gouty patient may drink the purely saline waters of Cheltenham," says the same authority, "with almost certain prospect of advantage." Indeed, our observations on the diseases relieved by the German waters of the same class, apply to those of Cheltenham. In one important particular the German waters differ from and excel the English, viz., in being impregnated with carbonic acid gas.

Leamington, in Warwickshire, is superseding Cheltenham as a fashionable spa; both places, however, are even more resorted to as residences for idle people than for the medicinal virtues of their springs. The Leamington waters are saline aperients: common salt, glauber salt, muriate of lime, and magnesia are the principal ingredients, varying much in the different springs; some contain iron and sulphuretted hydrogen gas. When taken in sufficient quantity the saline water acts as an aperient without pain or uneasiness. The diseases for which it is suited are similar to those of Cheltenham, and to the class of saline aperients generally.

Scarborough was once much resorted to for its mineral waters; the chief ingredients being sulphate of magnesia and a small quantity of iron. It acts on the bowels and kidneys.

With the exception of Carlsbad, the springs above described are cold; all of them are used internally, and all belong to the same class of mineral waters. They all act sensibly on the body by increasing the secretions from the intestines and kidneys; and they owe their active properties to saline salts—chiefly sulphate of soda or muriate of soda, combined with a small quantity of iron. We next arrive at the springs which are hot—thermal or volcanic waters, which are used chiefly as baths, but are also taken internally. The first we shall describe are very similar in their chemical contents to the above springs.

B. *Hot saline waters.* WIESBADEN, in Nassau, is more frequented by the English as a medicinal spa than any other. It may be reached in three or four days from England. The principal spring (the *Kochbrunnen*) is a hot, salt water; the principal ingredient being common salt, with which it is very strongly impregnated: temperature, 158° to 160°. It is principally frequented by the gouty and the rheumatic. "In gout of long standing, of the atonic kind, with or without deposition of calcareous matter in the joints, occurring in persons beyond the middle period of life, these baths are calculated to render the most eminent service." (Lee.) The stimulating nature of the water renders it more indicated in torpid and phlegmatic habits than for the more highly plethoric. Where there is much nervous irritability, with gout and rheumatism, the milder hot baths of Vichy, Ems, and Töplitz are preferable. The use of the douche in chronic local affections resulting from gout and rheumatism, adds much to the efficaciousness of the baths. Most chronic rheumatic affections are benefited. "Those depending upon long exposure to wet or cold, to which military men on duty are subject, are especially relieved by these baths." (Lee.) The water taken internally increases the secretions from the bowels and kidneys, and would be therefore useful in those cases where gout and rheumatism are combined with abdominal plethora, or congestion or irritation of the gastro-intestinal mucous membrane. It must be on this account that it relieves hypochondriasis, for the cure of which it is highly extolled.

BADEN-BADEN is in the neighbourhood of Weisbaden, and has hot salt springs of much the same quality. Both places are provided with all kinds of facilities for gambling. "A very few visits to the wells in the morning," says Dr. Johnson, "the hells in the evening, and the hotels in the middle of the day, will convince any observant traveller that three fourths of the sojourners at Baden go there to drink wine rather than water; and to lose money, rather than to regain health." (p. 93.)

C. *Hot Sulphurous Waters.* AIX-LA-CHAPELLE. The temperature of the hot sulphurous water is between 135° and 115° of Fahr. (Wetzlar), "the taste most nauseous, exactly resembling the washings of a gun-barrel, with a dash of rotten egg," (Dr. Johnson); but to this the palate soon becomes reconciled. Besides containing some sulphuretted hydrogen gas, it has nitrogen and carbonic acid gases in considerable quantities. It is highly impregnated with common salt, and also with carbonate of soda, besides some sulphuret of sodium. It is used externally as well as internally. The douche baths are admirably managed.

Its great reputation is for the cure of skin diseases, chronic rheumatism, secondary syphilitic and mercurial diseases, paralysis from lead and other mineral poisons. (Wetzlar.)

Barèges, on the French side of the central Pyrenees, is the most generally celebrated hot sulphurous spring, and very much frequented by the French. It is situated 4190 feet above the sea, and the village is only inhabited during the summer season; that is, from June to September. It is crowded, and lodgings are very difficult to be procured, and very expensive. Mr. Carmichael, of Dublin, who was at Barèges in 1829, was obliged to wait a fortnight before he could have the douche bath, and then to take it at eleven at night, as the only disengaged hour. The temperature is from 100° to 135°. The taste is nauseous, with the rotten-egg odour pertaining to sulphuretted hydrogen, but it is almost exclusively employed as baths. When taken internally it is stimulating and diuretic, but not aperient. When used as baths Mr. Carmichael found it a very powerful remedy. His disease was obstinate sciatica, connected with or dependent upon chronic indigestion, with probably gall-stones. The douche bath in a quarter of an hour produced feverishness, followed by loss of flesh. It is therefore a remedy contra-indicated in the plethoric or inflammatory, or where there is great nervous irritability, and is alone suited to the chronic forms of disease in the more torpid and less excitable temperament. Great numbers of paralytic persons resort to it. "Partial paralysis depending on some affections of the spinal cord; various neuralgic affections; chronic lumbago and sciatica; chronic rheumatism, and gouty affections of the joints; various cutaneous diseases, such as lepra, psoriasis, &c., are the complaints which have a fair chance of receiving benefit from these powerful waters." (Carmichael.) Their great utility in old, exfoliating gun-shot wounds has been so well established, that it is many years since the French government founded a military hospital into which all appropriate cases are sent for three successive summers. But if the patient after this trial is not found fit for duty, he is either pensioned or discharged from service.*

The Bagneres de Luchon, in the same district, are somewhat similar; and there are other springs among the Pyrenees which contain sulphur. The chemical composition, however, of the mineral waters of this district, as well as of the rest of France, has not been generally ascertained, and much ignorance consequently exists respecting their nature and use.

d. *Hot alkaline waters.* Vichy (Department de l'Allier,) has been very celebrated for its hot alkaline springs; the principal ingredients in which are bicarbonate of soda and carbonic acid gas. Struck with their utility in cases of gravel, and pondering on the connexion between gravel and gout, M. Petit, the resident physician at Vichy, has, in the last seven years, recommended them in gout with success. Previously to this time they were not employed for gout, and an older resident physician in the same place considered them to be injurious. In consequence of this difference of opinion the Royal Academy of Medicine in Paris appointed a commission to investigate the point. The reporter is M. Patissier, one of the members of the Academy, who had formerly expressed an opinion against the efficacy of these waters in the cure of gout. As much care seems to have been taken to arrive at the truth as is possible in such an investigation, and the report bears marks of that methodical arrangement and clearness of statement which belong so peculiarly to our clever

* R. Carmichael; Dublin Medical Journal, May, 1838.

clear-headed French neighbours. In this respect it is a contrast to some of the visionary, one-sided, obscure, exaggerated eulogies which are produced by spa-doctors generally, and by many of the German ones especially. But in saying this we should also add, that the report is an exception to the general carelessness of the French scientific men in regard to their own mineral waters. Very few of them have been analysed; and although the government and the Royal Academy of Medicine have drawn up excellent schemes for statistical returns, and the French system of centralization seems to offer the means of carrying their purpose into effect, yet the apathy and want of candour of the medical men who reside at the springs render all their plans abortive. As, however, this report contains important data for estimating the effects of hot alkaline waters on gout, and the inferences are applicable to all waters of a similar character, we shall take advantage of it.

M. Petit furnished the Academy in proof of his views with eighty cases of gout, not selected, but taken indiscriminately, being all of those who had taken the waters regularly, had followed up temperate habits, had persevered for some time in alkaline treatment, and had subsequently passed over two winters—the season in which attacks of gout are most frequent. Those cases which occurred only the year before the commission was issued were not included, as sufficient time had not elapsed to prove the efficacy of the waters; neither were those cases mentioned of patients who only imperfectly followed the treatment: for it is not probable that a constitutional disease such as gout could be radically cured by the temporary use of any mineral water whatever. To gain all the information possible, the reporter visited all those patients who were in Paris; and the secretaries of the Academy wrote to those who lived in the departments a series of questions: the greatest number answered them; and it is chiefly from the answers which are in accordance with the cases and observations of M. Petit that the cases have been prepared by the reporter, and the deductions drawn. Thus it will be evident that no pains have been spared to arrive at the truth in this report, and although perfect accuracy is impossible, yet that it must be a valuable document.

The deductions here drawn will of course apply not only to Vichy, but to all other warm alkaline springs, such as Ems, Töplitz, and even the less alkaline hot mineral waters which are charged with carbonic acid gas, as the Wildbad, Schlangenbad, &c. The differences in these waters, practically, is perhaps more in degree than in kind; and the variety of degrees is so great, that the medical adviser has a considerable range, and can accommodate the water to the peculiarity of the case. Thus, if circumstances render it doubtful whether the more powerful ones are suitable, the less active waters may be used without fear.

Of the 80 cases furnished by M. Petit, there were 19 in which the gout did not return (at least during two years) after the use of the Vichy baths and waters, and following them up with the constant employment of alkaline drinks, (℥j of bicarbonate of soda in a pint of water,) and a suitable diet, namely, by avoiding acids, highly azotized food, (such as the stronger meats,) and spirituous liquors. There were 51 cases in which the same treatment has rendered the fits of gout less frequent, shorter, and less painful; and 10 cases in which these mineral waters seem to have been injurious. All were cases of articular gout, either acute or chronic. In 11 of the cases acute fits of gout came on whilst the patients were using the waters; but the attacks were less acute and shorter than under ordinary circumstances, and the feverish condition was considered to be no contra-indication. From a further analysis of the cases, the particulars of which it is unnecessary to give, the waters appear to have the same sanative influence on hereditary as on acquired gout, in long standing as in recent cases; and when it coexists with gravel it is more easily relieved. The power of these waters is very limited in removing the consequences of gout in the joints, such as contractions, nodosities, chalk-stones, or rigidities from anchylosis. Thus it seems that, of the whole 80 cases, 70 have been more or less benefited; the greatest number of the patients, who previously walked with some difficulty, experienced at least one or two attacks every year, and suffered almost constantly, have either had no return of gout during two years, or their attacks have been fewer, shorter, and less frequent: in all, the general health was improved, and they regained the strength, flesh, and cheerfulness they had lost for some time; and, finally, notwithstanding the disappearance of the gout, they have not experienced any consecutive disorder. The remaining ten patients suffered from symptoms which some of the physicians attributed to the alkaline treatment and others considered as independent of it. On this account we have read carefully the ten cases, in order to form our own opinion from them alone. Three of these (72, 73, 80,) are obviously inconclusive either way. In 3 the waters produced intestinal irritation, marked by indigestion (71,) diarrhœa (72,) and hemorrhage from the bowels; but one of these patients was a hard drinker, and the chronic diarrhœa produced by the waters was decidedly beneficial, whilst the other 2 drank the waters in immoderate quantities. In the 4 remaining cases the waters seemed decidedly to have disagreed. In one they produced (to use the patient's own words) a flow of blood to the head, and this repeatedly, so that he discontinued them. The reporter seems dissatisfied with the expression,—a proof of the strength of his own cerebral circulation. One of the most frequent primary effects of even the purest mineral waters is a sensation of fulness of the head and slight vertigo, particularly in the debilitated or the highly nervous. In the second case (a lawyer of sedentary habits and delicate constitution), these waters seem to have repeatedly produced severe headaches. In the third (a bad case of rheumatic gout, of long standing), they produced great debility; and in the fourth (a man of apoplectic make, of soft fibre, and great nervous susceptibility,) they seemed to have aggravated his gouty symptoms, which terminated eventually in apoplexy.

The reporter concludes with an opinion, that the number of cases are sufficient to prove that the waters of Vichy, followed up with alkalis, may be used with success in articular gout affecting the joints, and that they are preferable to other antiarthritic remedies, both from the facility of their administration and the little inconvenience which attends their employment. The 10 unsuccessful cases do not, we admit with the reporter, militate against this opinion; but they show that the effects of these waters should be carefully watched, and that the patients should not indulge in very large quantities to gratify their own tastes or views, as the water certainly exerts a powerful influence on the economy.

We shall quote a few of M. Petit's remarks, as they illustrate the action of this class of waters. The alkaline waters and baths of Vichy diminish the secretions from mucous membranes. Constipation is a frequent effect; chronic catarrhal discharges, chronic diarrhœa, and chronic catarrhus vesicæ (when no organic cause—as stone—exists,) are diminished, their character is changed, and they become natural. "Je ne sais que dire de l'influence des eaux de Vichy sur la *génération.* A Vichy, comme à presques toutes les eaux minérales, il vient de temps en temps des femmes avec la seule pensée d'y acquérir la faculté de devenir mères. Chez quelques unes, cela a réussi; chez la plupart, cela n'a rien fait. Les eaux y sont-elles pour quelque chose? Je l'ignore." (p. 168.) M. Petit considers that a purely vegetable diet is the most rational one in gout, but as few will submit to it, it is useless to order it. He does not forbid meat, but recommends its moderate use, and the avoidance of the stronger and more exciting meats, as well as dishes prepared with spices and vinegar, and all acid fruits. He attaches most importance to the almost constant use of alkaline drinks, temperance in all things, and abstinence from wine, (at least from acidulated wine,) liqueurs, and irritating drinks. Gouty inflammation of an internal organ is a contra-indication to the baths, but not inflammation of the joints. M. Petit never applies leeches; they diminish pain for the time, but render convalescence tedious.

Many more cases of acute than of chronic gout came to Vichy, and, if there is any difference, the waters succeed better in the acute form; in the chronic the cure is slower. Those with erratic, anormal, irregular, or visceral gout (often the cause of many anomalous, painful, and distressing symptoms,) have generally derived much advantage from these waters, with a suitable diet and alkaline drinks. Under the head, "Gout," (p. 333,) we have alluded to the symptoms which M. Petit considers as indicative of this form of the disease.

The Vichy waters are contra-indicated in phthisis, and in diseases of the heart of any standing; in all cases of hemoptysis and in dropsy, not confounding this, however, with the œdema of gout. The rheumatic and neuralgic do not frequent Vichy.

MONT-D'OR, in France, is another hot alkaline spa in great reputation. It was celebrated for the cure of phthisis, but the greater exactness of modern pathology has proved that the cases in which it is really efficacious are those of chronic pulmonary catarrh, or chronic pneumonia, and it seems to act by producing a vigorous determination of blood to the skin, and consequently copious perspirations. M. Bertrand, the resident physician, directs the treatment, often watching the effect thermometer in hand, feeling assured that he shall relieve the disease if he can efficiently stimulate the surface. Hæmoptysis does not even deter him, if the patient is not of an irritable habit of body. Indeed all the cases which are most benefited are those in which there is torpidity rather than irritability.

EMS, in Nassau, is a bathing place much frequented by the English. Its springs are of warm alkaline water; their temperature from 83° to 115°. They contain principally bicarbonate of soda and carbonic acid gas, but they are not simply alkaline, as they also hold in solution some common salt. Ems is situated in a narrow valley with little shade, so that in summer it is a hot and relaxing place. The baths stimulate the skin and relax the nervous system. Their chief reputation is for bronchial affections. Sir James Clark recommends them in bronchial diseases affecting those whose constitutions are very delicate, where both the bronchial and abdominal mucous surfaces are in a state of irritation and congestion, or when the functions of the uterus are defective. The Ems waters may then be ordered as a short preparative to the more exciting springs of Carlsbad, Kissingen, and Marienbad. The situation as well as the somewhat relaxing effect of the waters are contra-indications in those affections where there is much relaxation of the system, and in persons who are better in cold bracing weather, or in dry exposed situations. "Ems," says Dr. Granville, "can never suit a hypochondriac, no matter from what functional disorder his unhappy state may arise. It never can suit such females as have suffered from repeated losses, have become blanched, weak, and shaky, and whose head feels heavy yet light, composed yet empty, always on the *qui vive*, yet oppressed."

Töplitz is a hot alkaline bathing spa, frequented by the gouty, rheumatic, and crippled. The temperature of the waters is from 114° to 122°, and they contain bicarbonate of soda and a very small quantity of carbonate of iron, enough to stain the baths. Carbonic acid gas is in very small quantities, and in this respect these waters differ considerably from those of Vichy and Ems. Töplitz is about sixty miles from Carlsbad, is frequented by the haut-ton of Germany, and is the cheapest watering place Dr. Granville ever visited.

There are two other springs much frequented, which are warm and very slightly alkaline, Schlangenbad and Wildbad.

Schlangenbad, in Nassau, is a slightly alkaline warm water, the temperature about 86°, containing a little carbonate of soda with some carbonic acid, giving a softness to the skin and a pleasant stimulus to the whole body. It was brought into fashion here by the celebrated "Bubbles from the Brunnens of Nassau."

Wildbad, which is in the neighbourhood, is a somewhat similar thermal water, its temperature from 88° to 99°. It contains a very small quantity of solid ingredients, of which muriate of soda is in the largest proportion; but it contains much more carbonic acid gas than the Schlangenbad.

The effect of these two baths seems to be to tranquillize the nervous system, and they are frequented by the gouty, rheumatic, and paralytic. Where, however, these diseases are connected with irritable dyspepsia, congestion of the gastro-intestinal mucous membrane, or congestion of the abdominal organs generally, these waters cannot be expected to produce much benefit.

E. *Simple unmineralized hot waters.* There is another class of hot and warm waters, whose efficacy is acknowledged but unexplained by their chemical composition, unless their virtue depends, as it may do, on their extreme purity and freedom from any foreign matter, together with their heat. They are purer waters than those commonly used as drink. Of this kind are the waters of Gastein and Pfeffers on the continent, and the Bristol Hot Wells, Buxton, Malvern, and others in England.

Pfeffers. In this place, situated in the Swiss Alps, (vividly described by Dr. Johnson,) are very pure waters, the temperature about 100°. "They have neither taste, smell, nor colour. They will keep for ten years, without depositing a sediment or losing their transparency." For six centuries they have been resorted to by invalids—those affected with rheumatism, neuralgia, glandular swellings, and skin diseases—not by the merely fashionable spa frequenters, and their reputation is still great. "In many they produce slight vertigo, in more they act freely on the bowels."

Gastein, in Austria, among the Noric Alps, (that chain stretching between Munich and Vienna,) is another extremely pure hot water. Its temperature is about 118°, and it is unusually free from even the ordinary saline matters. It is rarely visited by the English. The fine mountainous situation of both these places must, without doubt, have much to do with the marvellous curative power they are said to possess over diseases of debility.

Buxton is in the wilder and more mountainous part of Derbyshire. Its water is warm and pure, temperature 82°, and the solid matter such as is found in every common spring. As a bath, the slight shock it produces is followed by a highly soothing and pleasurable glow over the whole body. Persons of delicate and irritable habits of body, and with parts weakened by disease, can generally bear this degree of cold, and overcome it by a very small reaction; to produce which appears to be a most salutary effort of the constitution. The cases most benefited are those in which a loss of action and sometimes even of perfect sensation has come on in particular limbs, owing to long or violent inflammation or external injury, where the just measure of action is past. Thus chronic rheumatism succeeding acute, especially in moving parts, is often wonderfully relieved. (Saunders.)

Bristol. The hot-well (so called) at Clifton is a fine clear tepid water, temperature 74°, containing no other solid matter than is found in almost all common springs, and in less quantities; but it is very agreeable to the palate, from its being slightly impregnated with carbonic acid gas. Its continued use, as might be expected, increases the flow of urine, and keeps the skin moist. It is but little employed now, but formerly it was greatly celebrated in consumption; and, as Dr. Saunders well observed, it might alleviate some of the most harassing symptoms of that disease, by moderating the thirst, the dry burning heat of the hands and feet, the partial night sweats, and the symptoms that are peculiarly pectoral. (Saunders, p. 125.)

Matlock, in Derbyshire, is a pure tepid water, temperature 66°. As a tepid bath it produces little shock, and is fitted for those delicate and languid habits that cannot exert sufficient reaction after an ordinary cold bath, which consequently produces headach, and lassitude. These baths may be employed in preparing the invalid or the delicate for the sea. The spot is strikingly beautiful.

f. *Simple mineralized hot waters.* There is still another class of warm waters, which have an undoubted effect on the system, and which chemistry does not explain: unlike those which we have mentioned they neither contain principles whose action upon the body is undeniable, nor are they of extraordinary purity. They contain various saline matters, which medically we consider of but little potency, but yet their action on the body is well marked. Such are the waters of

Bath, many of the warm waters of the Pyrenees, and of France, generally. Of course their heat alone will explain much of their effects, but not all; still their effects cannot be doubted.

Bath. This is the hottest spring in England, its temperature being 112° to 116°. Its solid ingredients are considerable, but chiefly consisting of the calcareous salts: sulphate of lime is the principal one, which seems to exert no very marked effect on the secretions. These render it a very hard water, unfit for domestic purposes. There is a very small quantity of iron, which is deposited quickly, as the water cools. When taken internally the Bath water quickens the pulse, produces a glow in the stomach, increases the heat of the body, and promotes the flow of urine. As a bath it stimulates the skin, and the whole body, and produces copious perspiration. Some years ago it was very generally recommended in cases where various anomalous symptoms of the head, stomach, and bowels were thought to depend on latent gout. The warmth of the baths often brought the disease to a crisis, by favouring its outward development, bringing on an acute attack (a fit) of gout in a joint. From its stimulating nature, and its tendency to produce constipation, it is contra-indicated in the plethoric and highly irritable habit of body; also whenever there is a disposition to inflammation, or where a quick pulse and dry tongue indicate a feverish disposition. In some chronic diseases affecting those of a torpid, phlegmatic or bilious temperament, such as gout, rheumatism, sciatica, lumbago, and various forms of palsy, and particularly in paralysis in the arms in painters from white lead, it is remarkably efficacious. In chlorosis the bath seems to quicken the languid circulation, and to stimulate the body very favorably; the small quantity of iron which the waters contain is, in such cases, of course, beneficial. Where gout, as so often happens, is connected with congestion of the mucous membrane of the stomach and bowels, with congestion of the liver and of the abdominal venous circulation, as in those who live very freely, who grow fat and large and full, and look high-coloured and red, or else unnaturally sallow, a water which acts on the skin and kidneys without increasing the abdominal secretions, cannot be very efficacious, and may, from its stimulating the whole system, be injurious. In such cases the waters of Cheltenham or Leamington are still better; the more active ones of Germany promise still more success.

Springs of the Pyrenees. In the Pyrenees, besides the sulphurous hot waters which we have before noticed, there are various warm and tepid saline springs much resorted to by the French: they vary in temperature from 80° to 122°, and contain the ordinary saline matters in but small quantities, so that their virtue must depend on their heat, and on the peculiar combination of their chemical ingredients. Mr. Carmichael visited *Bagnères de Bigorre* and found that, in his own case, by the use of the baths his appetite improved, that a painful feeling of distension after even slight repasts, which before existed, was removed, and that the secretions from his bowels and kidneys became more natural.

There are here also *Cauterets*, a very fashionable spa for Parisians, *St. Sauveur*, *Eaux Bonnes*, and *Chaudes*: they are all more or less celebrated in rheumatic and paralytic and cutaneous diseases; but little is known of their chemical composition, as the negligence and want of energy of French men of science, as far as regards their own mineral waters, are remarkable. One physician, who was sent there by government, gravely assures us that the analysis of all the springs of one spot (Bagneres) would occupy a laborious chemist for four or five years.—The scenery of the Pyrenees is of the finest kind. The spas are rather expensive places, as they are much crowded for a short time. Many of them are situated in valleys, or in other spots of rare beauty. Ten years ago Mr. Carmichael thought they were but little visited by the English, and so they are, comparatively, still; but travellers are directing their steps there much more frequently. Pau is now quite an English colony, and English habits and customs, as well as countenances, are known at the posting houses, inns, and hotels in that district of southern France. What amount of the supposed benefit derived from these baths depends on the fine, dry, elastic mountain air of this district, cannot be exactly determined, but that the climate has a great share in restoring the strength and spirits of the debilitated and nervous, we should judge from the experience of Mrs. Ellis, who resided fifteen months in that part of France, living at Pau in the winter, and among the Pyrenees in the summer, and who has given an account of her impressions of the place: "I do not recollect," she says, "once to have felt, during my whole residence in the south of France, that causeless and indescribable dejection of mind which most of the inhabitants of England at times experience, and with which no one is, perhaps, more intimately acquainted than myself: a want of elasticity and animal vigour from a sort of sinking of the soul which often makes the dawn of each successive morning appear like a renewal of a helpless conflict; every unexpected event a fresh hinderance to the course we have to pursue; and every necessary exertion an insupportable trial. In the south of France, supposing the mind to be free from the pressure of actual calamity, there is an effect produced by the clearness of the atmosphere, the brightness of the sunshine, and the elasticity of the air, which makes the mere animal sensation of being alive—of breathing and moving—a perpetual enjoyment."

Making all due allowances for the florid style of the lady, and for that love of sympathy which leads those of such tender organizations rather to overstate those feelings which call forth the pity of others than to confine themselves within that strict line which divides truth from fiction, yet, on the whole, we believe there is very much truth in these observations, and they strikingly show how many of the effects which, supposing the individual to have been undergoing a course of mineral waters in the same situation, would have been attributed to the waters rather than to the climate. Mrs. Ellis, however, it must be remembered, was not an actual invalid, but perhaps owed many of her uncomfortable sensations to her pen: whilst enlightening the ladies of England she was, perhaps, wasting her own *pabulum vitæ;* and whilst setting them to rights was introducing disorder and confusion into her own nervous system.

G. *Chalybeate Waters.* Iron is the principal medicinal ingredient of the waters of this class; it enters into the composition of most of the saline waters, but in so small a quantity as to be a useful, but not a predominating, principle. Thus, both in the saline springs of Carlsbad and Marienbad, there is iron, but their marked action is in increasing the secretions from the intestines and kidneys. Under the present head, we shall therefore mention only those springs which are direct tonics. These are Spa, Pyrmont,Schwalbach, and Marienbad, on the continent, and Tunbridge Wells in England.

Spa is a cold chalybeate water, with an abundance of carbonic acid gas, rendering it an agreeable beverage. The town of Spa is thirty miles south of Aix-la-Chapelle, and it may be reached the third day from London. It is a beautifully-situated place, more resorted to for pleasure than for health, and is provided with gambling houses. It was much frequented at one time, but two causes have thinned it: the "Bubbles of the Brunnen" sent the English to Nassau; and the separation of Belgium from Holland putting it out of fashion in the latter country, the Dutch have joined the English at Schwalbach.

Schwalbach, in Nassau, is a cold chalybeate charged with carbonic acid, rendering it a very agreeable drink.

Pyrmont, in Westphalia, is a strong chalybeate with a very large proportion of carbonic acid, and besides is a very hard water with much of the earthy carbonates. It has been thought to be "considerably rougher in its operation, and more active, than the water of Spa; and hence Hoffman concludes that it is peculiarly well fitted for the use of the Westphalians "who are in general of a robust constitution, and live upon hard strong food." It is certain that whatever effects are produced on delicate stomachs by a hard water, may be here apprehended from the large proportion of earthy salts.

All these waters highly charged with carbonic acid, produce a slight temporary feeling of intoxication. At Pyrmont, the country people flock to the springs, and many of them to enjoy this effect of the waters. For those whose stomachs are easily disordered, and are of great delicacy of constitution, Schwalbach would be a preferable tonic from its great facility of digestion. It contains more carbonic acid than the waters of Pyrmont. The waters of Spa are not hard like those of Pyrmont.

Marienbad. Two of the springs, the Carolinen-brunnen and the Ambrosius-brunnen, are highly-carbonated chalybeates, the former very analogous to the chalybeate of Schwalbach. Its distance would, therefore, prevent the English, who can get a similar water so much nearer, from visiting Marienbad simply for its chalybeate waters.

Bruckenau and Bocklet, near Kissingen in Bavaria, are both highly-carbonated chalybeates. Dr. Granville says, the Bruckenauer is considered to be "the clearest and most spirituous of all the known carbonated chalybeate springs." They are conveniently situated for those patients who, after taking the active salt waters of Kissingen, require a tonic to complete their cure.

A tonic water is often advantageous after a saline, and it so happens that, at many of the saline spas, there are chalybeate springs either at the same spot or in the neighbourhood. Bruckenau and Bocklet are in the proximity of Kissingen. At Marienbad, there are direct chalybeate springs, as well as saline aperients. Those who have used the saline waters of Carlsbad can easily take a chalybeate course at Marienbad or Franzensbad, which are both in the neighbourhood; and patients who have undergone a course of bathing at Ems or Wiesbaden, can fortify themselves by the pleasant chalybeate of Schwalbach.

Furnas. The only *hot* chalybeate spring we are aware of is in the valley of the Furnas in St. Michael's, one of the islands of the Azores. It is used as a bath by the Azoreans. It is a clear, sparkling, hot water; the carbonic acid which it contains holds the iron in perfect solution, but it is deposited in cooling in a thick bright orange crust. In the same valley are hot springs slightly alkaline, of the heat of boiling water, and a cold highly-carbonated chalybeate, like that of Schwalbach in Nassau. (Bullar's Azores.)

TUNBRIDGE WELLS. The water of Tunbridge is a very simple one as to the nature of its solid contents, having a small quantity of iron held in solution by carbonic acid. The quantity of carbonic acid, as well as iron, is very small in comparison with the German chalybeates of Spa, Pyrmont, Schwalbach, or Marienbad; in certain classes of complaints this is an advantage.

NITON. At the back of the Isle of Wight, near the village of Niton, there is a strong chalybeate spring containing a large quantity of iron and alum. It is one of the most powerful chalybeates known, but has not attracted much general notice.

Besides these there are, in very many parts of England, chalybeate waters of various kinds, the knowledge and use of which are completely local. Dr. Granville has described several spas in Yorkshire and elsewhere, which are resorted to by those living in their vicinity, and to his book we must refer those who are locally interested in them.

H. *Waters containing Iodine and Bromine.* Sea water, as is well known, contains hydriodic and hydro-muriatic acids in small quantities. Traces of iodine have been also detected in the chalybeate of Schwalbach, and of bromine in the springs of Kissingen and Wiesbaden. Recently, however, both iodine and bromine have been ascertained to exist in some German springs in quantities which are considered sufficient to render them powerful agents in the cure of scrofula.

CREUZNACH, on the river Nahe, which joins the Rhine at Bürgen, not far from Mayence, is the iodine spa of Germany. Salt for many years has been made in the immediate neighbourhood from salt springs, and the principal ingredients in the water of Creuznach are common salt and muriate of lime. The iodine and bromine are in very small quantities in the water which is drunk; thus five or six tumblers, or a pound, of the water contain only 0·27 grains of bromide of magnesia, and 0·003 grains of iodide of magnesia, but at the neighbouring salt-works the bittern (the fluid remaining after the common salt has crystallized) is more highly impregnated with iodine. In one analysis nearly five grains of iodine were found in sixteen ounces of bittern; and in another, twenty-six grains of iodide of soda existed in the same quantity of bittern. This bittern is added to the baths. If used at first in any quantity it produces irritation and eruptions on the skin, but when added gradually in the greater number of cases it produces after some length of time, eruptions, boils, or livid spots, with relief. The water of Creuznach if taken in any quantity, increases the number of stools. It is resorted to for all forms of scrofula; and, says Vetter, it will be celebrated as long as "glands and lymphatics continue to be affected, mucous membranes to be diseased, and glandular textures to be obliterated or infiltrated with pure albumen."

At HALL in Upper Austria, the water is more strongly impregnated with iodine, "according to some as much as 5·53 grains of it in sixteen ounces of water, though Fuchs states it to be but a third or a fourth part of that quantity." This *for centuries* has been resorted to by the scrofulous. Latterly however it has been deserted for

Ischl, in Upper Austria, the situation of which is extremely beautiful. The bittern contains a considerable quantity of brome. Steam-baths are constructed above the salt-works and are said to be very efficacious helps to the cure.

To Woodhall, an iodine spa in England, allusion has already been made.

*Rules for the Drinking of Mineral Waters.* There are certain general rules common to most spas as to the quantity, time, and season, in which their waters ought to be taken; but as there are exceptions, the patient should comply with the customs of the spa which he frequents.

The water is taken before breakfast; occasionally the dose is repeated in the afternoon. The most favorable time of the year is thought to be from May to September. The ordinary length of the course is from a month to six weeks; but two or more months may be necessary, and perseverance for many successive years. It would be inconsistent to expect that a chronic disease, depending upon causes which had been in operation for years, and which had disarranged the whole system, and perhaps disorganized some important organ, could be cured by any treatment of short duration. Abernethy's aphorism, "a chronic remedy for a chronic disease," should in all such cases be borne in mind by the medical practitioner, and duly impressed by him on his patient. The following judicious observations by Marcard we quote, as generally applicable to the treatment of all diseases of long standing, as well as from its special application to the present question. He is discussing the treatment of chronic diseases of the abdominal viscera. "Whatever are the remedies chosen to remove obstructions, whether saline salts of various kinds, or whey or vegetable juices, or soft and attenuating drinks, or the mineral waters of Carlsbad, Ems, Pfeffers, and similar ones of the same deobstruent character; or lavements, often so efficacious; or finally tepid baths; whatever remedy has been selected with judgment, it should be used a long time, and assiduously. Neither days nor weeks are sufficient; months are necessary, and when the diseases are inveterate, the same remedy should be persevered in for years."*

The quantity of water, and the length of the course, must be often regulated by the effects produced. If the water cures by evacuations from the bowels, the stools should be constantly examined, for the same reason that every careful physician makes it a point (or should do so), to examine the stools of a patient undergoing a course of aperient medicine, in order to regulate the quantity of the remedy and the duration of the treatment by the effects produced on these secretions. But in many patients (and this effect has been observed from mineral waters of all kinds), after the water or baths have been taken and used during two or three weeks without any very visible effect, a febrile state is set up, attended with uneasiness, hot and dry skin, bitter mouth, disturbed nights, a series of symptoms called the "crisis," the "bath-storm," the "point of

* Description de Pyrmont, traduite de l'Allemand de Marcard, tom. ii. p. 39.

saturation" (the body being then supposed to be saturated with the water), and the theory is that nature is exerting herself to throw off the disease. It seems agreed by all that such symptoms are, on the whole, favorable, and that they are often followed by copious evacuations, and with great relief to the disease. But when these symptoms occur, the patient should either diminish the quantity of water, or discontinue it. The only safe way, however, seems to be to consult the bath-physician.

The glasses used in Germany contain five or six ounces of water : the patient begins by drinking two or three glasses at a quarter of an hour's interval, and increases the number gradually, according as he can digest it or according to the effect it produces. If the first glass disagrees with the stomach, no more should be taken on that day; if cold water disagrees, it may be warmed. Persons of delicate habits are often obliged to take the water at first warm, and gradually to diminish its temperature; sometimes it is necessary to dilute the water with milk, whey, gum, or rice water. Exercise after drinking the water is beneficial by promoting perspiration. Dr. Bertrand (of Mont d'Or), cautions his patients against taking a second course in the same season. He has observed, like others, that the benefit is often felt some time after the water has been discontinued, and he thinks that this secret and beneficial action of the economy may be interfered with, and prevented by a second course.

Injury is constantly done by drinking mineral waters in excessive quantities ; the poor especially often think they cannot drink too much. At Vichy, M. Petit begins by giving patients who are very susceptible two or three glasses of three ounces each daily ; but in inveterate cases of chronic disease, he gradually increases the quantity to ten, twelve, or even fifteen glasses daily. Many gouty patients have taken as much as twenty or twenty-five glasses a day; and one case is mentioned in which the patient, of his own accord, is said to have drunk as much as eighty-four glasses in twenty-four hours! (Rapport, page 180.)

We had intended to have described the mud and gas baths, and have entered into some details as to the rules for using baths in general, deduced from experience at the German spas. We must, however, defer this until another time, and shall conclude with a brief notice of the diet which is recommended to those who visit these watering places. A table of diet is given in each book describing the medical virtues of a spa. The one which we select as most accordant with our own practical views of diet in ordinary chronic diseases, is that furnished by Professor Von Ammon of Dresden, and published by Dr. Granville.

*Drinks.* Strong and new beer, all heating wines and liqueurs, mulled wine and punch are forbidden ; the Germans also disapprove of tea and lemonade.

Cocoa, small quantities of coffee and chocolate, milk, but not as a usual drink, table-beer, barley-water, sugared water, and negus, at table are admissible.

*Vegetables.* Asparagus, peas, spinach, young beans, and cauliflowers when young, in small quantities, are allowed ; also wood-strawberries in moderation.

Potatoes, all new fruits, (as apples, apricots, gooseberries, melons, medlars, quinces, raspberries, pears, and plums,) all green salads, cucumbers, radishes, sorrel, as well as cabbages, carrots, onions, mushrooms, and truffles, are interdicted.

*Of farinaceous food.* Bread and oatmeal groats are allowed, but all pastry is forbidden.

*Fish.* Tender fish is allowed; salmon, eels, lamprey, cray-fish, red and pickled herrings, and anchovies, are forbidden.

*Poultry and Game.* Chicken and fowls, hare, partridges, pigeons, venison, and eggs, may be eaten, but not ducks or geese.

*Meat.* Every kind of meat, except pork and salted and smoked meats, is permitted; all animal fat is interdicted. Cheese, spices, and all ices, are forbidden.

The ordinary dietetic precepts, of great moderation as to quantity, eating no more than can be bòrne without a sensation of distension and oppression, abstaining totally in the intervals between meals, resting completely for at least half an hour after each meal, are to be observed, and the patient should be warned to abstain carefully from any article of diet, (although it may be found among permitted food,) that has formerly disagreed with him. Coffee, according to Dr. Heidler, is found to be particularly injurious to those who have abdominal congestions, particularly in those of an inflammatory habit, producing in such, uneasiness, palpitation, oppression, trembling of the hands, &c. Tea, says the same physician, without exception is injurious to all persons whose nerves are feeble, more or less according to its quality, quantity and the habits of taking it. Water is the best common drink, then well-fermented but not strong beer, or water mixed with a pure and not an acid wine; or such wine may be taken undiluted, if the patient has been accustomed to it, and is not heated by it.

Daily and moderate exercise according to the strength, without producing great heat or fatigue. The patient should retire to bed at ten, and rise at five or six; six or seven hours' sleep is the usual time required; too much sleep weakens the body and the mind. Sleeping after dinner as it tends to produce congestion of the head and chest, should be forbidden to those disposed to apoplexy and hydrothorax.

In concluding an article which has been extended considerably beyond our intentions when commencing it, we must observe that the action of mineral waters upon the body in various forms of disease, is a subject well worthy the attention of the medical practitioner, both from its affording a chance of cure in many chronic complaints, and from its guiding him in the choice and adaptation of remedies. Its study is calculated to give comprehensive and satisfactory views of the common origin of a large number of diseases, and to lead to their judicious treatment from its bringing most prominently forward the paramount necessity of attending with minute care to the diet, exercise, and habits of life, of all patients who are submitted, for the cure of complaints of long standing, to a course of medicine.

